# Dietary habits associated with growth development of children aged < 5 years in the Nouna Health and Demographic Surveillance System, Burkina Faso

**DOI:** 10.1186/s12937-020-00591-3

**Published:** 2020-08-09

**Authors:** Isabel Mank, Alain Vandormael, Issouf Traoré, Windpanga Aristide Ouédraogo, Rainer Sauerborn, Ina Danquah

**Affiliations:** 1 Heidelberg Institute of Global Health (HIGH), Medical Faculty, Heidelberg University, Heidelberg, Germany; 2 Centre de Recherche en Santé de Nouna (CRSN), Institut National de Santé Publique (INSP), Nouna, Burkina Faso; 3 Institut Universitaire de Formations Initiale et Continue (IUFIC), Université Ouaga II (UO2), Ouagadougou, Burkina Faso

**Keywords:** Child growth, Stunting, Wasting, Dietary diversity, Dietary pattern, Burkina Faso

## Abstract

**Background:**

Knowing which dietary habits are associated with child growth could lead to better long-term health outcomes and improve the design of food-based interventions. We aimed to identify dietary habits that are associated with the growth development of children aged < 5 years living in rural Burkina Faso.

**Methods:**

This study used cross-sectional baseline data from 514 children (8–59 months) within the Nouna Health and Demographic Surveillance System (HDSS) in 2018. Household socio-demographics and child dietary habits, height and weight were assessed. We constructed scores for dietary diversity (DDS) and food variety (FVS), and extracted exploratory dietary pattern scores (DPS) using principal component analysis (PCA). Child growth was measured using height-for-age (HAZ) and weight-for-height z-scores (WHZ). We used multiple-adjusted linear regressions considering for socio-economic factors to quantify associations.

**Results:**

In this study population (median 36 ± 14 months old), stunting (HAZ < − 2) was seen in 26% and wasting (WHZ < − 2) in 7%. The DDS (median 7 ± 2 food groups) was positively associated with WHZ, while the FVS (median 13 ± 8 food items) was inversely associated with HAZ. We identified 4 dietary patterns: leaves-based, beans and poultry-based, maize and fish-based, and millet and meat-based diets. Only the maize and fish-based diet showed a statistically significant and here positive trend for associations with WHZ.

**Conclusion:**

Growth development of children aged < 5 years continues to be a health problem in the Nouna HDSS. A higher dietary diversity and food variety and dietary patterns characterized by maize and fish and beans and poultry intake appear to be beneficial for growth of young children in this area.

## Background

Poor dietary habits of children aged < 5 years old may lead to delayed mental and motor development, increase the risk of morbidity and mortality in childhood, and have negative health implications into adulthood [1]. Considering demographic and socio-economic factors, a lack of food and nutrient intake is among the most important risk factors of poor child growth [2]. Infants and young children need a variety of foods to cover their nutrient demands to grow physically and mentally. Dietary diversity is often associated with an improved micronutrient and energy intake (calories and proteins), and thus, an improved nutritional and health status [3–7]. Knowing which dietary habits are associated with child growth could lead to better health outcomes and improve the design of food-based interventions.

However, there is a need to better understand the association between dietary habits and child growth. Child growth development can be measured by height-for-age (HAZ) and weight-for-height (WHZ) z-scores. We use these scores to define stunting at HAZ < − 2 and wasting at WHZ < − 2 [8–10]. Currently, the literature is controversial about the effects of food intake, dietary patterns and dietary diversity on child growth. Although there are no recommendations on a specific dietary diversity with regard to number of food groups (dietary diversity score = DDS) or food items (food variety score = FVS) that should be consumed by children, it is recognized as a key indicator for diet quality [4, 6]. Hence, higher DDS and FVS are positively associated with energy, fat, protein, carbohydrates and various vitamins and minerals [4]. As a complementary approach to diet quality, the actual combinations of food intake may provide an in-depth understanding of eating habits and might serve as additional predictors of child nutritional status [11]. Such exploratory dietary patterns (DPs) reflect the complexity of dietary behavior and provide a realistic impression on the overall diet structure [12–14]. A combination of diet-related indicators, such as DD, FV and DPs, will support a better understanding of the relationship between diet and child growth.

The results on the association of dietary diversity on stunting are mixed across regions and should be interpreted with caution. Previous research [2] found significant associations between dietary diversity score tertiles and HAZ, even when controlled for socio-demographic variables. In their review of 11 heterogonous DHS sites and several other international studies, the association seemed to be robust. However, in a study in Sri Lanka with children aged 6–59 months, DDS was positively associated with HAZ, but not with stunting, wasting or underweight defined as < − 2 SD [15]. In Ethiopia, however, DDS was not significantly associated with HAZ among children under 5 years of age [14]. In contrast, Sié et al. [16] found that greater DDS was significantly associated with increased HAZ and reduced stunting, but not with wasting in north-west Burkina Faso (children aged < 5 years).

In this study, we looked beyond single macro- and micronutrient deficiencies and nutritional diseases. Rather, we addressed the full range of dietary habits including dietary diversities and food varieties in relation to child growth. Impacts on child growth are multifactorial [8, 17]. Thus, we aimed to characterize dietary behavior and to determine the associations of dietary habits with HAZ and WHZ among children aged < 5 years living in rural Burkina Faso, while accounting for a wide range of contributing factors.

## Methods

### Study site

A rural site in Burkina Faso was chosen for the study due to its large burden of child undernutrition [10, 18]. According to the Ministry of Health in Burkina Faso [19] and the Food and Agricultural Organization (FAO) [20], 27.3% of the children aged < 5 years in 2016 were stunted (− 7.8% since 2009; mean HAZ − 1.31) and 7.6% wasted (− 3.7%; mean WHZ − 0.54) across the whole country. Our study was conducted in the Nouna Health and Demographic Surveillance System (HDSS), which is located 400 km to the North-West of the capital city Ouagadougou. The Nouna HDSS lies in the Sudano-Sahelian climatic zone with an annual rainfall of around 800 mm, and a single rainy season from May to October [21]. Small-scale subsistence farming is the main type of livelihood with the main food crops being sorghum, millet, maize, fonio, rice, beans and peanuts, and the main cash crops being sesame and cotton. The region is strongly affected by seasonal food insecurity with the highest prevalence of undernutrition from June to September [22].

### Study design, sample size, and procedures

#### Study design

Cross-sectional baseline data were collected from August through September 2018 among households with children between 8 and 59 months of age, which we abbreviate to children aged < 5 years. During the study period, the HDSS did not collect household information on children younger than 8 months. Local field workers were trained in conducting household interviews in the local languages. Direct verbal translation of the interviews was necessary. Data was collected on the characteristics of the child, the mother, the household head and the household. Households were defined as independent socio-economic units living in the same compound and joining resources to meet basic dietary and other vital needs. Only one person is recognized as the head of the household [23].

#### Sample size

Children aged < 5 years and the respective households were selected using a multi-stage stratified random sampling approach. The principle aim of the Nutrition & Climate Change (NutriClim) study was to link weather on a small-scale to child undernutrition defined by their growth. We identified 32 villages that were clustered around 5 local weather stations. From these villages, a total of 18 villages were then randomly sampled proportional to population size. As we carried out the survey in a population under continuous demographic surveillance since 1992 [23], we were able to select households who had at least one child < 5 years of age at the time of the survey. This led to 18.7% of all households covered by the Nouna HDSS. We estimated a stunting rate of 30% among children aged < 5 years during the lean season based on a previous survey conducted in the same study population [24] and set a design effect of 1.7. This led to a final sample size of *N* = 514 children aged < 5 years. Selected households who refused to take part in the survey or refused to give consent were replaced by households in the same village and with a child of a similar age.

#### Study procedures

##### Exposure: dietary behavior

We used two methods for dietary assessment among children (< 5 years) in the sampled households. Trained interviewers administered a single 24-h Dietary Recall (24 h DR), which captures the number of meals per day, the time of consumption, and the food item combinations. The recalls were answered by the mothers or primary caregivers on a random day of the week, weekend, or atypical day (e.g. local festivities). Trained fieldworkers also administered a culturally adapted semi-quantitative Food Frequency Questionnaire (FFQ), which assessed the child’s food intake frequency over the preceding 7 days. This FFQ listed locally available food items in pre-defined food categories and portion sizes. Locally adapted questionnaires, documentation and planning materials were kindly provided by Martin-Prével et al. [25] and Becquey et al. [12].

##### Outcome: Child anthropometry

We measured the recumbent length (Seca 417; measuring range 10-100 cm) of all children, who met one of the following three criteria: (i) unable to stand up, (ii) below 24 months old and/ or (iii) shorter than 85 cm. For all other children < 5 years, we measured the standing height using a stadiometer (Seca 213; measuring range 20-205 cm). Weight was measured using tared weighing scales (Seca 878; measuring range up to 200 kg, uncalibrated) following WHO standards [26–28]. Anthropometric measurements were taken twice by trained study personnel. The mean of the measurements was used for analysis. Anthropometric data were entered and analyzed using the WHO Child Growth Standards R igrowup package. This program compares weight-for-height (WHZ) and height-for-age (HAZ) of the child with the WHO reference population of children showing ideal physiological growth under optimal environmental conditions and independent of ethnicity, socio-economic status or type of feeding [27]. Accordingly, stunting and wasting were assigned a z-score of below − 2 for the same age and sex [9, 27].

##### Covariates: socio-demographic characteristics

Children have better health and nutritional adequacy when households have greater income and resources, leading to a more diverse diet, better access to health care, and improved environmental conditions [29, 30]. Thus, based on existing knowledge on the interactions of demographic and socio-economic factors with dietary habits and nutrition status [2, 8, 17], we included the following covariates in our analysis: the child’s sex and age in months, number of siblings aged < 5 years, breastfeeding status of the index child, diarrhea and other diseases during the previous 2 weeks, mothers’ and household heads’ education and ethnicity, and household wealth. Household wealth was calculated using the international wealth index (IWI) [31], which is easily reproducible and comparable across nations and regions. The IWI is preferred over other wealth index calculations when income and expenditure information or price values of assets are not available, as is the case in our study population. Principal component analysis (PCA) was applied with 11 household assets that were weighted on the first component and entered into the IWI formula. The index included (1) consumer durables (television, refrigerator, mobile phone, car, bicycle), (2) expensive assets (motorbike and DVD player), (3) cheap assets (radio and plow), (4) housing characteristics (quality of floors and toilet facilities), and (5) public assets (electricity access and quality of the water source). The IWI was divided into quintiles based on its ownership of household assets with 1 being the poorest and 5 being the wealthiest quintile.

### Ethical considerations

The study was conducted in accordance with the most recent version of the ethical principles of the Declaration of Helsinki, which is applicable for national and international regulatory requirements. Ethical approval was obtained both from the Heidelberg University Ethical Committee (S-180/2017) and the Nouna Health Research Center Ethical Committee. Parents of severely undernourished children (z-scores below − 3) were encouraged to visit the closest health care center for consultation. We provided a referral document with financial support for the visit. All caregivers of our study participants provided informed written consent.

### Statistical analysis

#### Data management and handling of missing data

EpiInfo version 3.5.3 software was used for data entry, which was undertaken independently by two persons to reduce data entry errors. Data were checked for correctness by a third person in case of incongruent entries. Data quality checks, data cleaning and statistical analyses were performed with StataIC version 15.0 and R version 3.4.3. Information on the child was checked with regard to sex and birth date based on the birth date in the Nouna HDSS, the reported age by the mother and the age written in the health card. In case of doubt, the measurements of the child were excluded from the analysis. The data were checked for missing and implausible information. Anthropometric measurements were excluded if the difference of the two measurements per child was implausible or if computed z-scores were biologically impossible as defined by the WHO [32]. Due to missing values for socio-demographic variables (*n* = 20), multiple imputation was used for the regression analyses.

#### Descriptive statistics

Demographic, socio-economic, clinical, and anthropometric characteristics were presented for the total study population and by sex. The mean and standard deviation (SD) were used for continuous variables with normal distribution, median and interquartile range (IQR) for non-normally distributed variables, and percentage and sample size (n) for categorical data.

#### Construction of DDS and FVS, and identification of dietary patterns

For the construction of the Dietary Diversity Score (DDS) and the Food Variety Score (FVS), we used FFQ data from the preceding 7 days. The DDS was expressed as the number of consumed food groups (FG), while the FVS was the count of consumed food items (FI) [4, 7]. Supplementary Table 1 shows how we allocated the food items to food groups guided by the FAO approaches [6]: (1) cereals, starchy roots, tubers and their products; (2) pulses, nuts, seeds and their products; (3) vegetables; (4) fruits, (5) vitamin A-rich fruits and vegetables; (6) flesh meat; (7) fish and seafood; (8) oils and fats; (9) milk and milk products; and (10) eggs. The number and nature of the food groups were identical for each age group in order to assure comparability of results. Remaining items such as sweets and beverages were not considered for the DDS and FVS due to their low nutrient content, but included to describe dietary patterns. For the latter, grouping was based on similarities in nutrient content and culinary use leading to 30 food groups. DDS and FVS were split into tertiles [2]. The tertile categories ranged from low (< 7 FGs or < 11 FIs), through average (7 FGs or 11–15 FIs) to high (> 7 FGs or > 15 FIs), with the lowest tertile being the reference. There is no international consensus on the assignment of food groups to the DDS [6, 29, 33, 34].

Exploratory dietary pattern scores (DPS) were identified through PCA using the intake frequencies of food items as assessed by the FFQ [13, 35]. PCA is a dimension-reduction technique that uses the correlation structure of intake frequencies to identify underlying food combinations [35, 36]. Food items were excluded from PCA when they were never consumed by more than 96% of the children. For missing values on the 7 day recall, we assumed that the child consumed this food item at least once per week if all other fields for the food item were filled out. In order to derive dietary patterns, an orthogonal rotation (varimax) was applied to ensure that the factors remained uncorrelated. The criteria to extract the optimal number of patterns comprised an eigenvalue of > 1.5. The scree plot in supplementary Figure 1 shows the number of factors or dietary patterns that should be generated by the analyses [37]. The proportion of explained variance in food intake for each component was set to a minimum of 5%. This yielded 4 identified factors explaining 32% of the total variance (supplementary Table 2). Foods with absolute factor loadings of above 0.40 were considered as major contributors to the pattern score. Each child received a factor score for the dietary patterns based on the factor loadings for the specific food item consumed and based on the frequency the child consumed this food item over the preceding 7 days. This showed the adherence of each child to the respective dietary pattern. Each DPS was then split into tertiles [14] to assess the distributions of socio-economic characteristics and food intake frequencies across the tertiles. We tested for trends across tertiles using the median values of the DPS-tertiles as the exposure variables.

#### Associations between dietary habits and nutritional status

We set up linear regression models to quantify the association of the DDS, the FVS, and the DPS (the predictor variables) with HAZ and WHZ (the outcome variables). Then, we investigated the assumption for linear relationships by calculating beta-coefficients and their 95% confidence intervals (CIs) for tertiles 2 and 3, with tertile 1 as the reference category. Model 1 shows the crude associations. In Model 2, we adjusted for age and sex of the child, and study area. In Model 3, we additionally adjusted for mothers’ and household heads’ education and ethnicity, household wealth (IWI), the number of siblings aged < 5 years, diarrhea incidence and sickness of the child over the previous 2 weeks, and breastfeeding status. We assessed the robustness and the clinical relevance of our findings in a sensitivity analysis, using the binary outcomes stunting and wasting. Poisson regression models were used and the results presented as prevalence ratios with 95% CIs. The same set of adjustment variables were added as described above.

## Results

### Sample description

Table 1 displays the characteristics for the sample and by sex of the child. Boys and girls were equally distributed (boys: 50%) with a similar mean age (36 ± 14 months). The mothers had a mean age of 30 ± 7 years, were mainly illiterate (78%) and housewives (92%). The household heads were primarily illiterate (75%), worked as farmers (85%), and about a third lived in polygamous marriages (37%). The households reported a mean of 12 ± 6 household members and 3 ± 2 children aged < 5 years. The children in the sample had a mean birth weight of 3056 ± 520 g, 12% had a low birth weight (< 2500 g), and 40% were exclusively breastfed until 6 months of age. Diarrhea was present in 19% and 41% had a history of acute illness in the past 2 weeks. The mean height and the mean weight of the children were 90 ± 10 cm and 12 ± 3 kg, respectively. Stunting was discernible in 26% and wasting in 7% of the children.

**Table 1:** General characteristics of children aged < 5 years (*n* = 514) by sex in the Nouna HDSS

Characteristics N		Nouna HDSS		Boys		Girls	
		%	n	%	n	%	n
		100.00	514	49.61	255	50.39	259
**Demographics**							
Childrens’ age^a^	Months	35.87	13.90	36.17	13.64	35.57	14.18
Region	Toni	46.69	240	46.67	119	46.72	121
	Cissé	17.12	88	14.90	38	19.31	50
	Kodougou	11.87	61	13.33	34	10.42	27
	Nouna	14.40	74	15.29	39	13.51	35
	Sono	9.92	51	9.80	25	10.04	26
**Socio-economics**							
Mothers’ age^a^	Years	29.56	6.83	29.36	6.71	29.16	6.96
Mothers’ education	Illiterate	78.00	397	78.97	199	77.04	198
	Literate	4.91	25	5.56	14	4.28	11
	Primary	13.36	68	12.70	32	14.01	36
	Secondary	3.73	19	2.78	7	4.67	12
Household heads’ education	Illiterate	74.56	381	71.94	182	77.13	199
	Literate	12.13	62	13.83	35	10.47	27
	Primary	10.96	56	11.07	28	10.85	28
	Secondary	2.35	12	3.16	8	1.55	4
Mothers’ ethnicity	Dafing	30.41	156	30.98	79	29.84	77
	Bwaba	22.61	116	22.75	58	22.48	58
	Mossi	20.47	105	20.39	52	20.54	53
	Peul	20.47	105	19.22	49	21.71	56
	Other	6.04	31	6.67	17	5.53	14
Household heads’ ethnicity	Dafing	30.35	156	30.59	78	30.12	78
	Bwaba	22.96	118	22.75	58	23.17	60
	Mossi	18.09	93	18.04	46	18.15	47
	Peul	22.76	117	22.35	57	23.17	60
	Other	5.84	30	6.27	16	5.40	14
Mothers' marriage	Polygame	34.50	177	34.90	89	34.11	88
Household heads’ marriage	Polygame	36.77	189	36.08	92	37.45	97
Mothers’ occupation	Housewives	92.16	470	93.31	237	91.02	233
Household heads’ occupation	Farmers	84.86	426	87.95	219	81.82	207
Wealth index (IWI)	Poorest	21.57	107	23.08	57	20.08	50
	Poor	18.75	93	23.48	58	14.06	35
	Middle	19.56	97	19.03	47	20.08	50
	Rich	24.60	122	22.67	56	26.51	66
	Richest	15.52	77	11.74	29	19.28	48
Household size^a^		12.30	6.45	12.03	6.40	12.57	6.49
Siblings aged <5 years^a^		2.74	1.70	2.65	1.70	2.82	1.70
**Clinical**							
Birth weight^a^	Gramm	3056	520	3081	533	3031	506
Low birth weight	< 2500 g	12.99	53	13.17	27	12.81	26
Exclusive breastfeeding	Yes	38.91	200	40.78	104	37.07	96
Diarrhea the last 2 weeks	Yes	19.30	99	22.35	57	16.28	42
Child sick last 2 weeks	Yes	41.52	213	43.53	111	39.53	102
**Child anthropometry**							
Height^a^	cm	89.72	10.11	90.52	9.53	88.94	10.61
Weight^a^	kg	12.37	2.72	12.68	2.54	12.06	2.86
Height-for-Age (HAZ)^a^	Z-score	−1.27	1.34	−1.28	1.41	−1.26	1.27
Weight-for-Height (WHZ)^a^	Z-score	−0.47	1.02	−0.43	1.08	−0.51	0.96
Stunting	< −2 SD	25.64	131	27.38	69	23.94	62
Wasting	< −2 SD	6.68	34	7.14	18	6.23	16

^a^ Mean and SD

### Meal frequencies and dietary diversity

According to the 24 h DR data, the mean number of meals per day was 4 ± 1. The meal frequency was higher in younger children (8–23 months; 5 ± 1 meals) than in older ones (24–59 months; 3 ± 1 meals). If excluding children currently being breastfed, the mean number of meals per day was the same as for older children (3 ± 1 meals). About 91% of the children had an evening meal between 5 and 8 pm, and 20% consumed a meal at night (after 8 pm), which consisted mainly of maternal milk and other milk products. One in five children consumed cereals for their three main meals: early morning (before 9 am), lunch time (12-2 pm), and evening meal (5–8 pm) (supplementary Table 3).

**Fig. 1:**
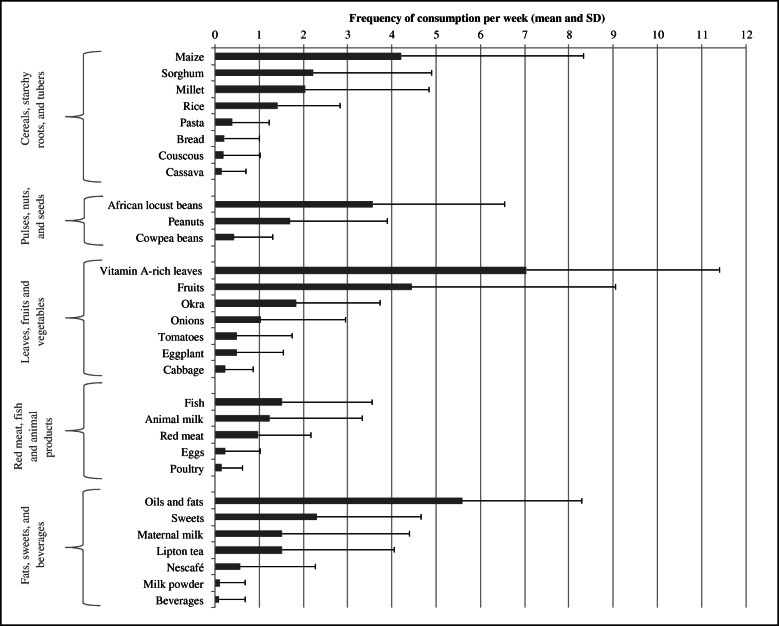
Intake frequencies (mean and SD) of servings per week for 30 food items among children aged < 5 years (*n* = 514) in the Nouna HDSS

According to the 7-day FFQ, the median DDS was 7 ± 2 food groups and the median FVS was 13 ± 8 food items. There was no trend across DDS- (*p* = 0.344) or FVS-tertiles (*p* = 0.662) by sex. The children were older in higher tertiles of both indices as compared to the lower tertile of each score (DDS: 35 months versus 37 months of age, *p* = 0.128; FVS: 34 months versus 38 months of age, *p* = 0.007). 99% of the children consumed cereals, starchy roots, tubers and their products at least once per week. This was followed by Vitamin A-rich leaves (94%), oils and fats (92%), pulses, nuts, seeds and their products (85%), vegetables (79%), fruits (61%), flesh meat (59%), milk and milk products (54%), fish and seafood (49%), and eggs (10%). Although this order did not change much between age groups, older children consumed more animal products (meat, fish and eggs), whereas the youngest group (8–23 months) had higher intakes of milk and milk products, including breast milk (supplementary Table 4). Out of possible 117 food items, 36 were eaten by the children during the time of the lean season. Figure 1 displays the mean weekly frequency of the 30 food groups consumed by the children. The figure shows that maize, leaves, and oils and fats were consumed the most. Maize is likely to be prepared as a porridge (*tô)* with a sauce made out of leaves. Fish or meat may be added to the sauce, if available. Other food groups such as beverages (e.g. coca cola), milk powder, animal products, cabbage and cassava were seldom consumed. More specifically, Baobab leaves were eaten the most (80% of the children), followed by shea butter (78%), rice (70%), African locust bean/soumbala (68%), okra (62%), roselle leaves (59%), roselle fruit and dry maize (each 55%), and sorghum (51%) with at least half of all children consuming these food items. For infants (8–23 months), the order of food groups stayed the same, though 79% consumed mother’s milk (*N* = 133).

### Dietary patterns by children aged < 5 years

Four distinct dietary patterns (DPS) were identified: “leaves-based diet”, “beans and poultry-based diet”, “maize and fish-based diet”, and “millet and meat-based diet” (supplementary Table 2). The leaves-based diet positively correlated with cabbage, vitamin A-rich leaves, peanuts and onions. The beans and poultry-based diet showed high loadings for cowpea beans, bread, poultry, pasta, milk powder and sweets. The maize and fish-based diet was characterized by frequent intakes of fish, okra, eggplant and maize. The millet and meat-based diet was characterized by millet and flesh meat. Maize showed a negative factor loading for the latter and thus was negatively associated with this DPS. Supplementary Table 5 presents the characteristics of the study population across tertiles of the identified DPS and the frequency the food items were consumed over a week with respect to the specific dietary pattern. Children in lower tertiles were younger compared to children in higher tertiles, with the exception of the millet and meat-based diet which showed no difference across tertiles. In the last tertile of the maize and fish-based diet, there were more girls as compared to the first tertile. This was the opposite for the beans and poultry-based diet.

### Associations of dietary indices with HAZ and WHZ

Tables 2 and 3 present the associations for DDS, FVS and the four DPS divided by HAZ and WHZ. The results of the crude model show that the association of DDS and FVS with HAZ was negative. These were attenuated after accounting for demographic factors (Model 2) and socio-economic and clinical factors (Model 3). Only the association of FVS with HAZ was statistically significant in the multiple-adjusted Model 3, where HAZ decreased by 0.02 (95% CI: − 0.05, 0.00, *p* = 0.047) for a 1 score-point increase in FVS. With regard to dietary patterns, HAZ was inversely associated with higher leaves-based (− 0.03, 95% CI: − 0.04, − 0.01, *p* = 0.003) and millet and meat-based DPS in the unadjusted models (not-significant). Apart from the crude model of the leaves-based diet none of the associations of HAZ with the dietary patterns were statistically significant.

**Table 2:** Associations of DDS, FVS and four DPS with HAZ of children aged < 5 years (*n* = 514) in the Nouna HDSS

Height-for-Age z-score (HAZ)		Per 1 score-point increase			Tercile 1	Tercile 2		Tercile 3	
		β-coef.	95% CI	p-value trend		β-coef.	95% CI	β-coef.	95% CI
**Dietary Diversity Score (DDS)**									
Model 1	Crude model: HAZ	−0.06	− 0.12, 0.01	0.074	Ref.	−0.50	− 0.80, − 0.20	−0.13	− 0.40, 0.14
Model 2	Model 1 + demographics	− 0.02	−0.09 0.04	0.489	Ref.	−0.44	−0.76, − 0.12	−0.04	− 0.34, 0.27
Model 3	Model 2 + socio-economics + clinical	− 0.04	−0.11, 0.03	0.314	Ref.	−0.37	−0.69, − 0.05	− 0.12	− 0.42, 0.17
**Food Variety Score (FVS)**									
Model 1	Crude model: HAZ	−0.03	−0.05, − 0.01	0.005**	Ref.	− 0.46	−0.76, − 0.16	−0.43	− 0.71, − 0.15
Model 2	Model 1 + demographics	− 0.02	− 0.05, 0.00	0.057	Ref.	−0.41	− 0.73, − 0.09	− 0.33	− 0.65, 0.00
Model 3	Model 2 + socio-economics + clinical	− 0.02	− 0.05, 0.00	0.047*	Ref.	−0.40	− 0.71, − 0.09	− 0.37	− 0.69, − 0.04
**Leaves-based dietary pattern score**									
Model 1	Crude model: HAZ	− 0.03	− 0.04, − 0.01	0.003**	Ref.	− 0.21	− 0.50, 0.08	− 0.42	− 0.70, − 0.14
Model 2	Model 1 + demographics	−0.02	− 0.04, 0.00	0.094	Ref.	−0.14	− 0.43, 0.15	− 0.29	− 0.61, 0.03
Model 3	Model 2 + socio-economics + clinical	0.00	−0.02, 0.02	0.807	Ref.	0.05	−0.24, − 0.41	−0.07	− 0.41, 0.26
**Beans and poultry-based dietary pattern score**									
Model 1	Crude model: HAZ	0.00	−0.03, 0.03	0.788	Ref.	−0.10	−0.40, 0.19	0.02	−0.28, 0.31
Model 2	Model 1 + demographics	0.00	−0.03, 0.04	0.817	Ref.	−0.09	−0.38, 0.20	0.08	−0.24, 0.41
Model 3	Model 2 + socio-economics + clinical	−0.01	− 0.04, 0.02	0.490	Ref.	−0.14	− 0.42, 0.14	− 0.08	− 0.40, 0.24
**Maize and fish-based dietary pattern score**									
Model 1	Crude model: HAZ	0.01	−0.02, 0.03	0.522	Ref.	0.00	− 0.30, 0.30	0.00	− 0.28, 0.29
Model 2	Model 1 + demographics	0.00	−0.03, 0.03	0.993	Ref.	− 0.05	−0.36, 0.26	− 0.10	− 0.42, 0.21
Model 3	Model 2 + socio-economics + clinical	0.00	− 0.03, 0.02	0.909	Ref.	−0.24	− 0.54, 0.06	− 0.15	− 0.46, 0.17
**Millet and meat-based dietary pattern score**									
Model 1	Crude model: HAZ	−0.01	− 0.04, 0.01	0.272	Ref.	−0.05	−0.32, 0.22	− 0.30	−0.59, 0.00
Model 2	Model 1 + demographics	0.00	−0.03, 0.02	0.779	Ref.	0.06	−0.21, 0.33	−0.18	− 0.49, 0.13
Model 3	Model 2 + socio-economics + clinical	0.02	−0.01, 0.05	0.155	Ref.	0.19	−0.09, 0.46	0.07	−0.25, 0.39

* p-value < 0.05, ** *p*-value < 0.01

**Table 3:** Associations of DDS, FVS and four DPS with WHZ of children aged <5 years (n = 514) in the Nouna HDSS

Weight-for-Height z-score (WHZ)		Per 1 score-point increase			Tercile 1	Tercile 2		Tercile 3	
		β-coef.	95% CI	p-value trend		β-coef.	95% CI	β-coef.	95% CI
**Dietary Diversity Score (DDS)**									
Model 1	Crude model: WHZ	0.07	0.02, 0.12	0.005**	Ref.	0.26	0.03, 0.48	0.29	0.08, 0.50
Model 2	Model 1 + demographics	0.05	0.00, 0.11	0.058	Ref.	0.23	−0.01, 0.47	0.25	0.04, 0.47
Model 3	Model 2 + socio-economics + clinical	0.06	0.00, 0.12	0.043*	Ref.	0.28	0.05, 0.52	0.29	0.06, 0.51
**Food Variety Score (FVS)**									
Model 1	Crude model: WHZ	0.02	0.00, 0.03	0.027*	Ref.	0.25	0.02, 0.47	0.27	0.04, 0.49
Model 2	Model 1 + demographics	0.01	−0.01, 0.03	0.213	Ref.	0.22	− 0.02, 0.45	0.18	− 0.06, 0.43
Model 3	Model 2 + socio-economics + clinical	0.01	−0.01, 0.03	0.252	Ref.	0.26	0.02, 0.49	0.20	−0.05, 0.46
**Leaves-based dietary pattern score**									
Model 1	Crude model: WHZ	0.01	0.00, 0.02	0.219	Ref.	0.09	−0.13, 0.31	0.02	−0.19, 0.23
Model 2	Model 1 + demographics	0.00	−0.01, 0.01	0.920	Ref.	−0.01	−0.24, 0.22	− 0.07	−0.30, 0.15
Model 3	Model 2 + socio-economics + clinical	0.01	−0.01, 0.02	0.506	Ref.	0.07	−0.18, 0.32	−0.01	− 0.26, 0.25
**Beans and poultry-based dietary pattern score**									
Model 1	Crude model: WHZ	0.02	0.00, 0.05	0.025*	Ref.	0.15	−0.07, 0.38	0.28	0.06, 0.50
Model 2	Model 1 + demographics	0.02	0.00, 0.04	0.068	Ref.	0.15	−0.07, 0.37	0.22	−0.01, 0.46
Model 3	Model 2 + socio-economics + clinical	0.01	−0.01, 0.04	0.244	Ref.	0.15	−0.08, 0.38	0.19	−0.06, 0.44
**Maize and fish-based dietary pattern score**									
Model 1	Crude model: WHZ	0.03	0.01, 0.05	0.001**	Ref.	0.31	0.08, 0.53	0.34	0.12, 0.56
Model 2	Model 1 + demographics	0.03	0.01, 0.05	0.007**	Ref.	0.21	−0.02, 0.45	0.29	0.04, 0.53
Model 3	Model 2 + socio-economics + clinical	0.03	0.01, 0.06	0.004**	Ref.	0.21	−0.03, 0.45	0.34	0.09, 0.60
**Millet and meat-based dietary pattern score**									
Model 1	Crude model: WHZ	−0.01	−0.03, 0.00	0.135	Ref.	0.07	−0.15, 0.30	−0.14	− 0.35, 0.07
Model 2	Model 1 + demographics	− 0.02	−0.04, 0.00	0.023*	Ref.	0.06	−0.16, 0.28	−0.25	− 0.47, − 0.02
Model 3	Model 2 + socio-economics + clinical	−0.01	− 0.03, 0.01	0.232	Ref.	0.08	−0.15, 0.30	−0.16	− 0.40, 0.08

* p-value < 0.05, ** p-value < 0.01

With regard to WHZ, there were positive associations with DDS and FVS in the crude models. These relationships were attenuated in Model 2, but still showed a positive and with DDS a significant association in the fully adjusted Model 3 (0.06, 95% CI: 0.00, 0.12, *p* = 0.043). Higher beans and poultry-based and a maize and fish-based dietary pattern scores showed a positive association with WHZ in the crude model before adjustments for demographic, socio-economic and clinical factors (Models 2 and 3) were made. A significant trend for associations with WHZ were only observed for the maize and fish-based DPS in the multiple-adjusted model (0.03, 95% CI: 0.01, 0.06, *p* = 0.004). All other dietary patterns were not significantly associated with WHZ in the fully adjusted model.

We also ran a sensitivity analyses using stunting and wasting as binary outcome variables (supplementary Table 6 and Table 7). For stunting, the prevalence increased by 3% (95% CI: 1.01, 1.06, *p* = 0.014) for a 1 score-point increase in DDS and by 3% (95% CI: 1.02, 1.03, *p* < 0.0001) for a 1 score-point increase in FVS in the fully adjusted Model 3. By contrast, the prevalence of wasting reduced by 7% (95% CI: 0.89, 0.97, *p* = 0.001) for 1 score-point increase in DDS and by 5% (95% CI: 0.94, 0.97, p < 0.0001) for a 1 score-point increase in FVS (Model 3). The beans and poultry-based and the maize and fish-based diets were associated with a 6 and 11% reduction in wasting, respectively after adjusting for socio-economic and clinical factors. There was a slight decrease in stunting for the maize and fish-based (1%) and the millet and meat-based diets (2%) in the fully adjusted models.

## Discussion

### Summary of main results

Our NutriClim study in the Nouna HDSS of 2018 found that children 8–59 months old had a mean HAZ of − 1.27 and a mean WHZ of − 0.47. On average, the children had 4 meals per day. 99% of them integrated cereals, starchy roots, tubers and their products into their meals, which they mainly consumed in the early morning (before 9 am), at noon (12–2 pm) and in the evenings (5–8 pm). Maize, leaves, and oils and fats were consumed the most. Vitamin A-rich leaves make up 94% (Baobab leaves) and oils and fats 92% (shea butter) of the children’s diet, with 10% consuming eggs. 79% of the younger children (8–23 months) consumed mainly mother’s milk. Children consumed a median of 7 out of 10 food groups, and 13 out of 36 food items over a week. DDS and FVS increased linearly with age.

Associations of HAZ and WHZ with dietary habits were in general not robust in the fully adjusted models. In the adjusted model for socio-economic factors, we observed a negative trend in HAZ with an increase in FVS and a positive trend in WHZ for an increase in DDS. Additionally, we showed that after adjustment for socio-economic factors, the associations between dietary factors and HAZ were mitigated. The same results were observed in the sensitivity analysis using stunting (HAZ < − 2) and wasting (WHZ < − 2) as binary outcomes. Four distinct dietary patterns featured by energy source were identified, labelled as “leaves-based diet”, “beans and poultry-based diet”, “maize and fish-based diet”, and “millet and meat-based diet”. Higher DPSs were associated with increasing age. We found that HAZ was negatively associated with the leaves-based diet in the unadjusted model, but not in the fully adjusted model or with any other dietary pattern. By contrast, WHZ was positively associated with the beans and poultry-based diet before adjustments for socio-economic factors and significantly associated with the maize and fish-based diets in the fully adjusted model. Socio-economic factors had a positive association with HAZ, but not with WHZ.

### Dietary habits among young children in the Nouna HDSS

The diet of children in the Nouna HDSS relies heavily on foods planted by the household and provided by nature. The main agricultural products in Burkina Faso are cereals (millet, sorghum, maize, rice, fonio), oil seeds (cotton, peanuts, sesame, niébé (beans), soy, voandzou); roots and tubers (igname, patate, manioc, potato); fruits and vegetables (mango, agrumes, tomato, onion, green beans); and sugar canes [38]. The highest portion of the diet is provided by locally produced maize and rice in the form of porridge and combined with a leaves-based sauce and shea butter that can be collected from the surrounding trees. Although only 39% of the mothers reported exclusive breastfeeding for the first 6 months after childbirth, children were on average breastfed until 23 months of age. This is below the global target to have 70% of children exclusively breastfed until 6 months of age by 2030 [39]. In low resource populations, especially from the developing world, diets are often mainly based on starchy staples with little or no animal products and few fruits and vegetables. Yet, plant-based diets tend to have a low quantity of micronutrients, with few being easily absorbed [4]. Subsequently, a closer observation of nutrient intake and uptake would be recommended in the study region.

Our results showed that the children had a median DDS of 7 out of 10 food groups, and a median FVS of 13 out of 36 selected and 117 selectable food items over a week. Other studies such as by Sié et al. [33] reported an average of 6 out of 11 food groups for children in the Nouna HDSS over a 7-day recall period, while Nikièma et al. [40] counted only 2 out of 9 food groups for children in the same age group in rural Houndé, Burkina Faso, and with only 25% meeting their minimum dietary diversity of at least 4 food groups. However, the latter recall was done only over the previous 24 h, confirming a low dietary diversity over a 24 h DR compared to over a week. Yet, although a high DDS is considered a good indicator for dietary adequacy [4], emphasis should also be placed on food and nutrient quantity and diversity [6]. This is often neglected in the literature. Our results showed that higher DDS and FVS of the children were mainly driven by more frequent intakes of energy-dense foods such as cereals, oils and fats, and beans (Fig. 1 and supplementary Table 4), which are essential for weight gain. Therefore, this might explain a positive relationship of DDS and FVS with WHZ. At the same time, foods rich in essential micronutrients (e.g. fruits and animal-based products), which are important for healthy linear growth, only partially contributed to DDS and FVS (Fig. 1 and supplementary Table 4). This may explain some of the inverse association of DDS and FVS with HAZ. Since dietary diversity cannot provide direct information on quantities and nutrient uptake, the association of DDS and FVS with HAZ and WHZ should be interpreted with caution [2].

### Associations between dietary habits and nutritional status

While dietary pattern analyses do not allow observing the effect of specific nutrients on disease risks, it does provide insight on the associations of the overall diet as a combination of food items [13]. Melaku et al. [14] found a positive association with HAZ for diets with a high intake of dairy, vegetables and fruits (ß-coef. = 0.19). All three food groups were hardly consumed by the children in our sample and did not meet the requirements to be assigned to a dietary pattern. Yet, loading scores for vegetables and fruits were higher in the beans and poultry-based and the maize and fish-based diets, which were not associated with HAZ, but with stunting as a binary outcome compared to the other two diets. A positive association between fish consumption and stunting among 6–23 months old children (OR = 0.947) was also found in Zambia by Marinda et al. [41]. Dairy consumption is especially important for children as it provides important nutrients such as protein, calcium and vitamin A for bone growth. Yet only every fifth child over 24 months of age in the Nouna HDSS reported to have consumed some form of milk (maternal or animal milk) (supplementary Table 4). Considering the lack of dairy, vegetables and fruits in the leaves- and millet- and meat-based diets, they might show important impacts on reducing child stunting. As we controlled for number of days a food item was consumed, the reliability of our results increased.

In our sample of children aged < 5 years, FVS was negatively associated with HAZ and DDS was positively associated with WHZ. This was also the case after controlling for various socio-demographic factors and when tested with stunting and wasting as binary outcomes. Accordingly, wasting and WHZ improved significantly with increasing DDS and FVS, while results for stunting and HAZ showed to be highest in the middle DDS tertile and worsened with increasing FVS (33% and − 1.60 SD). Similar results were reported by Arimond and Ruel [2] for Mali, a country bordering Burkina Faso. They reported that the middle DDS tertile compared to the lowest DDS had the highest mean HAZ (− 0.11 SD). On the contrary, a higher DDS was related with higher HAZ (0.23 SD), which we cannot confirm for our results.

However, global results on the association of DDS and FVS with HAZ and WHZ seem to be contradictory. Steyn et al. [5] reports that most studies showed positive correlations between HAZ and WHZ with DDS and FVS, with the exception of WHZ, which showed a negative correlation among children aged 1–3 years. Hatloy et al. [29] reported no correlation between WHZ and DDS and FVS in rural Mali. In another study in Sri Lanka among children aged 6–59 months of age, dietary diversity was positively associated with HAZ, but not with WHZ, or stunting and wasting as binary outcomes [15]. In Ethiopia, however, DDS was not significantly associated with HAZ among children under 5 years of age [14]. Yet, Sié et al. [33] report a positive association with stunting and DDS in contrast to wasting, which showed no association, as “dietary diversity scores are reflective of longer-term nutritional habits”. This assumption cannot be confirmed with our study since we show the opposite. Such results indicate the need for further, comprehensive research in the area of child nutritional status and dietary adequacy and to provide attention to single results.

### Nutritional status of young children in the Nouna HDSS

Child growth can also be defined by stunting (HAZ < − 2) and wasting (WHZ < − 2) as an indicator for children’s nutritional status. Stunting and wasting continue to be high among children aged < 5 years in the Nouna HDSS [24]. This is a critical time span to develop optimal health, growth and neuro-development [18, 19, 42]. During the lean season in the Nouna HDSS in 2018, 26% of the children were stunted (mean − 1.27 SD) and 7% wasted (mean − 0.47 SD), which are similar results for two study villages in the Nouna HDSS as conducted by Sié et al. [33] in July 2017. They reported 21% stunting and 10% wasting among children in the same age group. Yet, according to Beiersmann et al. [24], seasonal differences in child growth development need to be considered. They found that in June 30% and in December 45% of the children < 3 years were stunted in 2009. On the contrary, more children < 3 years were affected by wasting in June (26%) compared to December (16%) in the same year. The results mirror our own findings as the lean season represents low food stocks leading to acute undernutrition, while those affects can only be observed on chronic undernutrition once several months have passed [2, 43]. Hence, this might explain a lack of association between dietary diversity and stunting when measured at the same point in time. The dietary habits of the lean season might show even higher stunting prevalence in the Nouna HDSS a few months later.

### Public health research and policy implications

First, we would like to encourage further research on seasonal variations in diets and their link to child growth in order to even better measure the impact of diets on chronic undernutrition. Secondly, we observed a need for nutrition interventions and counselling that do not only provide solutions to continuous child undernutrition, but also prevent food insecurity and provide adaptation and copying mechanisms to a changing environment [11]. Thirdly, further research is needed on the macro- and micronutrient intake to receive a precise feedback on nutrient deficiencies and uptake in order to structure sustainable and successful interventions. In this regard and fourthly, nutrition-related research requires interdisciplinary approaches and cohort studies, which allow combining several aspects rather than looking at single impacts and to consider for temporal differences between seasons and years [40]. Lastly, by clustering the research area varying environmental impacts such as weather variability and extremes can be taken into account as an additional possible explanation for stagnating or even increasing child undernutrition and food insecurity (FAO et al., 2018; Nelson & International Food Policy Research Institute, 2010), which will be elaborated on in a subsequent paper. This will contribute to a grounded understanding of causes of and threats to undernutrition. We would like to encourage policy makers to specifically target stunting among children, to support the understanding of households and mothers on the importance of a nutritious and diverse diet bevor, during and after pregnancy, and to promote dietary diversity to further reduce and subsequently eliminate wasting.

### Strengths and limitations

Our study findings had several strengths and weaknesses. Firstly, in order to control for the association of DDS and FVS with HAZ and WHZ, we controlled for various socio-demographic factors. By doing so, we were able to emphasize the link and importance of dietary diversity, which is not very well represented in the literature and unique for our study area. However, our measures of socio-demographic factors might be imperfect and not complete. Secondly, the period of retrospective dietary assessments are greatly discussed in the literature as longer recall periods are assumed to be prone to missing information, which was limited in this study by applying a 24 h DR plus a 7-day FFQ.

Thirdly, we did not examine the relationship between food quantities, dietary diversity and child growth. We did not attempt to measure food quantities for the following reasons: portion sizes within this age group cannot be standardized; the time for such detailed assessment is burdensome for the study participants; foods are regularly consumed from shared bowls; and the low level of formal education of mothers limits the precision of recalls [30]. To better understand the relationship to food amounts, though, we conducted a desk review in Pubmed and google scholar (search terms in various combinations used: dietary diversity/ diversity, quantity/ amount/ food quantity/ calories, and HAZ/ stunting). Surprisingly, we did not find studies that examined this combination of indicators. Most studies focus on the use of nutrition indicators to measure the quality of the diet and the need to consider for energy and nutrient quantities, but do not report the amount of the foods consumed. Both, dietary diversity and dietary pattern scores, correlate positively with energy intake and thus, food amounts. Therefore, the link between portion sizes and dietary diversity as well as dietary patterns would be worth studying.

Fourthly, we were not able to control the association of dietary habits with stunting and wasting for different time-lags [43]. Somé and Jones [44] looked at household dietary diversity by 4 seasons in Burkina Faso in 2014. They found out that dietary diversity was significantly higher during the beginning of the lean season, during the lean season and the highest during the harvest seasons compared to the post-harvest season. The post-harvest season covers the time from around December to April, and which represents a possible 6 months prior to the time when the data collection for NutriClim took place. As Somé and Jones did not use anthropometric data in their analyses, further research looking at stunting and its lagged association with dietary diversity would be highly recommended.

## Conclusions

A higher dietary diversity and food variety and dietary patterns characterized by maize and fish, and beans and poultry intake appear to be beneficial for growth development of children aged < 5 years in the Nouna HDSS area. Yet, their integration into food-based interventions and possible sustained health effects need further investigations. As the temporal relationships between dietary exposure and nutrition outcomes are essential, we recommend accounting for a time-lag, specifically when looking at dietary diversity in correlation with child height.

## Supplementary information

**Additional file 1:.** Table 1 Overview of food groups and food items.

**Additional file 2:.** Figure 1 Scree plot of eigenvalues after factor analysis to derive dietary patterns.

**Additional file 3: **Table 2 Rotated factor loadings of food items for the four identified dietary pattern scores among children aged < 5 years (*n* = 514) in the Nouna HDSS. * Factor loading scores above 0.40 indicate relevant contribution to the pattern score.

**Additional file 4:.** Table 3 Percentage of 523 children aged < 5 years who consume FFQ-derived food groups according to the eating time of the day.

**Additional file 5:.** Table 4 Percentage of 523 children aged < 5 years who consume FFQ-derived food groups according to age group.

**Additional file 6: **Table 5 Characteristics and frequencies of food intake of children aged < 5 years (*n* = 514) across tertiles of dietary pattern scores: leaves-based diet, beans-based diet, maize-based diet, and millet-based diet. * *p*-value < 0.05, ** p-value < 0.01, *** p-value < 0.001. * p-value < 0.05, ** p-value < 0.01, *** p-value < 0.001. * p-value < 0.05, ** p-value < 0.01, *** p-value < 0.001. * p-value < 0.05, ** p-value < 0.01, *** p-value < 0.001.

**Additional file 7:.** Table 6 Sensitivity analyses of associations of DDS, FVS, and four DPS with stunting (HAZ < − 2) of children aged < 5 years (n = 514) in the Nouna HDSS. * p-value < 0.05, ** p-value < 0.01, *** p-value < 0.001.

**Additional file 8:.** Table 7: Sensitivity analyses of associations of DDS, FVS and four DPS with wasting (WHZ < − 2) of children aged < 5 years (n = 514) in the Nouna HDSS. * p-value < 0.05, ** p-value < 0.01, *** p-value < 0.001.

## Data Availability

The dataset used and analyzed for the NutriClim study is available from the corresponding author on reasonable request and with permission of the Nouna Health Research Center, Burkina Faso.

## Abbreviations

CI: Confidence Interval
CRSN: Centre de Recherche en Santé de Nouna
DDS: Dietary Diversity Score
DPS: Dietary Pattern Score
FAO: Food and Agricultural Organization
FFQ: Food Frequency Questionnaire
FVS: Food Variety Score
HAZ: Height-for-Age Z-score
HDSS: Health and Demographic Surveillance System
HIGH: Heidelberg Institute of Global Health
INSP: Institut National de Santé Publique
IWI: International Wealth Index
NutriClim: Nutrition & Climate Change
PCA: Principal Component Analysis
SD: Standard Deviation
UO2: Université Ouaga II
WHO: World Health Organization
WHZ: Weight-for-Height Z-score
24 h DR: 24-h Dietary Recall

## References

[1] Pan American Health Organization (PAHO), World Health Organization (WHO) (2003). Guiding principles for complementary feeding of the breastfed child.

[2] Arimond M, Ruel MT (2004). Dietary diversity is associated with Child nutritional status: evidence from 11 demographic and health surveys. J Nutr.

[3] Arimond M, Ruel MT. Progress in developing an infant and child feeding index: An example of using the Ethiopian demographic and health survey, FCND Discussion Paper No. 143. Washington, DC: International Food Policy Research Institute (IFPRI); 2002. Available from: https://www.ifpri.org/publication/progress-developing-infant-and-child-feeding-index.

[4] Ruel MT (2003). Operationalizing dietary diversity: a review of measurement issues and research priorities. J Nutr.

[5] Steyn N, Nel J, Nantel G, Kennedy G, Labadarios D (2006). Food variety and dietary diversity scores in children: are they good indicators of dietary adequacy?. Public Health Nutr.

[6] Food and Agricultural Organization (FAO), Kennedy G, Ballard T, Dop MC (2010). Guidelines for Measuring Household and Individual Dietary Diversity (reprint 2013).

[7] Sibhatu KT, Krishna VV, Qaim M (2015). Production diversity and dietary diversity in smallholder farm households. Proc Natl Acad Sci.

[8] Akombi B, Agho K, Hall J, Wali N, Renzaho A, Merom D (2017). Stunting, wasting and underweight in sub-Saharan Africa: a systematic review. Int J Environ Res Public Health.

[9] de Onis M, Dewey KG, Borghi E, Onyango AW, Blössner M, Daelmans B (2013). The World Health Organization’s global target for reducing childhood stunting by 2025: rationale and proposed actions: WHO global stunting reduction target. Maternal Child Nutrition..

[10] Victora CG, de Onis M, Hallal PC, Blossner M, Shrimpton R (2010). Worldwide timing of Growth faltering: revisiting implications for interventions. Pediatrics.

[11] Bukania ZN, Mwangi M, Karanja RM, Mutisya R, Kombe Y, Kaduka LU (2014). Food insecurity and not dietary diversity is a predictor of nutrition status in children within semiarid agro-ecological zones in eastern Kenya. J Nutrition Metabol.

[12] Becquey E, Savy M, Danel P, Dabiré HB, Tapsoba S, Martin-Prével Y. Dietary patterns of adults living in Ouagadougou and their association with overweight. Nutrition J. 2010;9(1) [cited 2019 Apr 25]. 10.1186/1475-2891-9-13. 10.1186/1475-2891-9-13 PMC2848625 20307296

[13] Hu FB (2002). Dietary pattern analysis: a new direction in nutritional epidemiology. Curr Opin Lipidol.

[14] Melaku YA, Gill TK, Taylor AW, Adams R, Shi Z, Worku A. Associations of childhood, maternal and household dietary patterns with childhood stunting in Ethiopia: proposing an alternative and plausible dietary analysis method to dietary diversity scores. Nutrition J. 2018;17(1) [cited 2019 Oct 24]. 10.1186/s12937-018-0316-3. 10.1186/s12937-018-0316-3 PMC5789646 29378583

[15] Perkins JM, Jayatissa R, Subramanian SV (2018). Dietary diversity and anthropometric status and failure among infants and young children in Sri Lanka. Nutrition..

[16] Sié A, Kynast-Wolf G, Zabré P, Winkler V, Müller O, Stieglbauer G, et al. Clustering of Infant Mortality Within Families in Rural Burkina Faso. Am J Tropical Med Hygiene. 2018; [cited 2018 Dec 14]. 10.4269/ajtmh.17-0669. 10.4269/ajtmh.17-0669 PMC6335915 30457090

[17] Black RE, Victora CG, Walker SP, Bhutta ZA, Christian P, de Onis M (2013). Maternal and child undernutrition and overweight in low-income and middle-income countries. Lancet.

[18] Poda GG, Hsu C-Y, Chao JC-J (2017). Factors associated with malnutrition among children <5 years old in Burkina Faso: evidence from the demographic and health surveys IV 2010. Int J Qual Health Care.

[19] Ministère de la Santé Burkina Faso, the United States Agency for International Development (USAID), United Nations Children's Fund (UNICEF), World Food Programme (WFP), World Health Organization (WHO), African Development Fund (ADF). Enquête nutritionnelle nationale 2016; 2016. p. 53.

[20] Food and Agriculture Organization of the United Nations (FAO), International Fund for Agricultural Development (IFAD), United Nations Children's Fund (UNICEF), World Food Programme (WFP), World Health Organization (WHO). The State of Food Security and Nutrition in the World2018. Building climate resilience for food security and nutrition. Rome: FAO; 2018. ISBN 978-92-5-130571-3.

[21] Diboulo E, Sié A, Rocklöv J, Niamba L, Yé M, Bagagnan C (2012). Weather and mortality: a 10 year retrospective analysis of the Nouna health and demographic surveillance system, Burkina Faso. Global Health Action.

[22] Dixon S, Holt J (2010). Livelihood Zoning and Profiling Report: Burkina Faso. A Special Report by the Famine Early Warning Systems Network. The United States Agency for International Development Famine Early Warning Systems Network (FEWS NET).

[23] Sié A, Louis Valérie R, Gbangou A, Müller O, Niamba L, Stieglbauer G (2010). The Health and Demographic Surveillance System (HDSS) in Nouna, Burkina Faso, 1993–2007. Global Health Action.

[24] Beiersmann C, Bountogo M, Tiendrébeogo J, Louis VR, Gabrysch S, Yé M (2012). Malnutrition in young children of rural Burkina Faso: comparison of survey data from 1999 with 2009: malnutrition in young children. Tropical Med Int Health.

[25] Martin-Prevel Y, Allemand P, Nikiema L, Ayassou KA, Ouedraogo HG, Moursi M (2016). Biological Status and Dietary Intakes of Iron, Zinc and Vitamin A among Women and Preschool Children in Rural Burkina Faso. van Wouwe J, editor. PLOS ONE.

[26] van Stuijvenberg ME, Nel J, Schoeman SE, Lombard CJ, du Plessis LM, Dhansay MA (2015). Low intake of calcium and vitamin D, but not zinc, iron or vitamin a, is associated with stunting in 2- to 5-year-old children. Nutrition..

[27] Child Growth Standards WHO (2006). Length/height-for-age, weight-for-age, weight-for-length, weight-for-height and body mass index-for-age: methods and development [internet].

[28] World Health Organization (WHO). Training course and other tools. Child growth standards. Geneva: WHO; 2008. Available from: https://www.who.int/childgrowth/training/en/. [cited 2017 Mar 23].

[29] Hatløy A, Hallund J, Diarra MM, Oshaug A (2000). Food variety, socioeconomic status and nutritional status in urban and rural areas in Koutiala (Mali). Public Health Nutr.

[30] Savy M, Martin-Prével Y, Traissac P, Delpeuch F (2007). Measuring dietary diversity in rural Burkina Faso: comparison of a 1-day and a 3-day dietary recall. Public Health Nutr.

[31] Smits J, Steendijk R (2015). The international wealth index (IWI). Soc Indic Res.

[32] World Health Organization (WHO) (2013). WHO Child Growth Standards R igrowup package.

[33] Sié A, Tapsoba C, Dah C, Ouermi L, Zabre P, Bärnighausen T (2018). Dietary diversity and nutritional status among children in rural Burkina Faso. Int Health.

[34] Swindale A, Bilinksy P (2006). Household Dietary Diversity Score (HDDS) for Measurement of Household Food Access: Indicator Guide (Version 2).

[35] Vankaiah K, Brahmam G, Vijayaraghavan K. Application of Factor Analysis to Identify Dietary Patterns and Use of Factor Scores to Study their Relationship with Nutritional Status of Adult Rural Populations. J Health Population Nutrition. 2011;29(4) Available from: http://www.banglajol.info/bd/index.php/JHPN/article/view/8448. [cited 2019 Aug 1]. 10.3329/jhpn.v29i4.8448 PMC3190363 21957671

[36] Balder H., Virtanen M, Brants HAM, Krogh V, Dixon LB, Tan F, Mannisto S, Bellocco R, Pietinen P, Wolk A, Berrino F, Van den Brandt PA, Hartman AM, Goldbohm RA. Common and country-specific dietary patterns in four european cohort studies. J Nutr. 2003;133(12):4246–51. 10.1093/jn/133.12.4246. 10.1093/jn/133.12.4246 14652380

[37] Frank LK, Kröger J, Schulze MB, Bedu-Addo G, Mockenhaupt FP, Danquah I (2014). Dietary patterns in urban Ghana and risk of type 2 diabetes. Br J Nutr.

[38] WFP, Ministere de Burkina Faso, Fewsnet. Burkina Faso. Analyse Globale de la Vulnérabilité, de la Sécurité Alimentaire et de la Nutrition (AGVSAN). Rome: WFP; 2014.

[39] Food and Agriculture Organization of the United Nations (FAO), International Fund for Agricultural Development (IFAD), United Nations Children's Fund (UNICEF), World Food Programme (WFP), World Health Organization (WHO). The State of Food Security and Nutrition in the World 2019: Safeguarding against economic slowdowns and downturns. Rome: FAO; 2019. Available from: 10.18356/fe66e08f-en.

[40] Nikièma L, Huybregts L, Martin-Prevel Y, Donnen P, Lanou H, Grosemans J (2017). Effectiveness of facility-based personalized maternal nutrition counseling in improving child growth and morbidity up to 18 months: A cluster-randomized controlled trial in rural Burkina Faso. van Wouwe JP, editor. PLOS ONE.

[41] Marinda PA, Genschick S, Khayeka-Wandabwa C, Kiwanuka-Lubinda R, Thilsted SH (2018). Dietary diversity determinants and contribution of fish to maternal and under-five nutritional status in Zambia. Wieringa F, editor. PLoS ONE.

[42] de Onis M, Branca F (2016). Childhood stunting: a global perspective. Maternal Child Nutrition.

[43] Brown ME, Grace K, Shively G, Johnson KB, Carroll M (2014). Using satellite remote sensing and household survey data to assess human health and nutrition response to environmental change. Popul Environ.

[44] Somé JW, Jones AD (2018). The influence of crop production and socioeconomic factors on seasonal household dietary diversity in Burkina Faso. Ahmad A, editor. PLOS ONE.

