# Supplementary material for: Dietary habits associated with growth development of children aged < 5 years in the Nouna Health and Demographic Surveillance System, Burkina Faso
Source: Nutr J. 2020 Aug 9;19:81. doi: 10.1186/s12937-020-00591-3 (PMC7416397; doi:10.1186/s12937-020-00591-3)
Supplement: Supplementary file 1 — Additional file 1:. Table 1 Overview of food groups and food items. [file 12937_2020_591_MOESM1_ESM.docx]

# Supplements

Table 1 Overview of food groups and food items

| **FG 1** | **DDS food groups** | **FI** | **FVS food items** | **FG 2** | **Input food group for factor analysis** | **Scientific rational** |
| --- | --- | --- | --- | --- | --- | --- |
| 1 | Cereales, starchy roots, tubers and their products | 1 | Banana plantain |  |  | Excluded, because >99% never consumed this item |
|  |  | 2 | Bread | 1 | Bread |  |
|  |  | 3 | Broken millet porridge | 2 | Millet | Food items combined |
|  |  | 4 | Millet (small) |  |  |  |
|  |  | 5 | Cassava | 3 | Cassava |  |
|  |  | 6 | Couscous | 4 | Couscous |  |
|  |  | 7 | Dry maize | 5 | Maize | Food items combined |
|  |  | 8 | Fresh maize |  |  |  |
|  |  | 9 | Maize porridge |  |  |  |
|  |  | 10 | Fonio |  |  | Excluded, because >98% never consumed this item |
|  |  | 11 | Pasta (macaroni) | 6 | Pasta (macaroni) |  |
|  |  | 12 | Potato |  |  | Excluded, because >99% never consumed this item |
|  |  | 13 | Rice | 7 | Rice |  |
|  |  | 14 | Sorghum | 8 | Sorghum |  |
|  |  | 15 | Yam tuber |  |  | Excluded, because >99% never consumed this item |
| 2 | Pulses, nuts, seeds and their products | 16 | African locust bean/ soumbala | 9 | African locust bean/ soumbala |  |
|  |  | 17 | Bambara groundnuts (voandzou) |  |  | Excluded, because >96% never consumed this item |
|  |  | 18 | Coconut |  |  | Excluded, because >99% never consumed this item |
|  |  | 19 | Cowpea beans (niébé) | 10 | Cowpea beans (niébé) |  |
|  |  | 20 | Palm seeds/ nuts |  |  | Excluded, because >99% never consumed this item |
|  |  | 21 | Peanut butter | 11 | Peanuts | Food items combined |
|  |  | 22 | Peanut flour |  |  |  |
|  |  | 23 | Peanuts |  |  |  |
|  |  | 24 | Sesame |  |  | Excluded, because >97% never consumed this item |
|  |  | 25 | Soya bean |  |  | Excluded, because >99% never consumed this item |
| 3 | Vegetables | 35 | Avocado |  |  | Excluded, because >99% never consumed this item |
|  |  | 36 | Cabbage | 12 | Cabbage |  |
|  |  | 37 | Cucumber |  |  | Excluded, because >99% never consumed this item |
|  |  | 38 | Garlic |  |  | Excluded, because >95% never consumed this item |
|  |  | 39 | Zucchini |  |  | Excluded, because >98% never consumed this item |
|  |  | 40 | Lettuce |  |  | Excluded, because >99% never consumed this item |
|  |  | 41 | Zucchini |  |  | Excluded, because >98% never consumed this item |
|  |  | 42 | Eggplant | 13 | Eggplant |  |
|  |  | 43 | Okra | 14 | Okra |  |
|  |  | 44 | Onions | 15 | Onions |  |
|  |  | 45 | Tomatoes | 16 | Tomatoes |  |
| 4 | Fruits | 26 | Papaya | 17 | Fruits | Food items combined |
|  |  | 27 | Roselle fruit |  |  |  |
|  |  | 28 | Shea fruit/ flesh |  |  |  |
|  |  | 29 | Sweet banana |  |  |  |
|  |  | 30 | Watermelon |  |  |  |
|  |  | 31 | Dates |  |  |  |
|  |  | 32 | Dattock (detar/ kagha) |  |  |  |
|  |  | 33 | Lemon |  |  |  |
|  |  | 34 | Tamarind fruit |  |  |  |
| 5 | Vitamin A rich fruits and vegetables | 46 | African locust bean fruit |  |  | Excluded, because >98% never consumed this item |
|  |  | 47 | Baobab leaves | 18 | Vitamin A rich leaves | Food items combined |
|  |  | 48 | Bay leaves |  |  |  |
|  |  | 49 | Cowpea bean leaves |  |  |  |
|  |  | 50 | Drumstick leaves |  |  |  |
|  |  | 51 | Jute leaves |  |  |  |
|  |  | 52 | Onion leaves |  |  |  |
|  |  | 53 | Roselle leaves |  |  |  |
|  |  | 54 | Spinach |  |  |  |
|  |  | 55 | Melon |  |  | Excluded, because >99% never consumed this item |
|  |  | 56 | Parsley |  |  | Excluded, because >99% never consumed this item |
|  |  | 57 | Pepper |  |  | Excluded, because >97% never consumed this item |
|  |  | 58 | Sweet potato |  |  | Excluded, because >99% never consumed this item |
| 6 | Flesh meat | 59 | Caterpillar |  |  | Excluded, because >99% never consumed this item |
|  |  | 60 | Chicken meat | 19 | Poultry |  |
|  |  | 61 | Goat meat | 20 | Red meat | Food items combined |
|  |  | 62 | Beef meat |  |  |  |
|  |  | 63 | Sheep meat |  |  |  |
|  |  | 64 | Pork meat |  |  |  |
|  |  | 65 | Guinea fowl meat |  |  | Excluded, because >97% never consumed this item |
|  |  | 66 | Rabbit meat |  |  | Excluded, because >99% never consumed this item |
| 7 | Fish and seafood | 67 | African carp | 21 | Fish | Food items combined |
|  |  | 68 | Carp |  |  |  |
|  |  | 69 | Catfish |  |  |  |
|  |  | 70 | Perch fish (Nil) |  |  |  |
|  |  | 71 | Sardine |  |  |  |
|  |  | 72 | Shiny-nose (capitaine) |  |  |  |
|  |  | 73 | Tuna |  |  |  |
| 8 | Oils and fats | 74 | Cottonseed oil | 22 | Oils and fats | Food items combined |
|  |  | 75 | Olive/ vegetable oil |  |  |  |
|  |  | 76 | Palm oil |  |  |  |
|  |  | 77 | Peanut oil |  |  |  |
|  |  | 78 | Shea butter |  |  |  |
| 9 | Milk and milk products | 79 | Animal milk | 23 | Animal milk |  |
|  |  | 80 | Milk powder | 24 | Milk powder |  |
|  |  | 81 | Mother's milk | 25 | Mother's milk |  |
|  |  | 82 | Yoghurt |  |  | Excluded, because >99% never consumed this item |
| 10 | Eggs | 83 | Chicken eggs | 26 | Eggs | Food items combined |
|  |  | 84 | Guinea fowl eggs |  |  |  |
| 11 | Sweets | 85 | Biscuit | 27 | Sweets/ sugar | Food items combined |
|  |  | 86 | Honey |  |  |  |
|  |  | 87 | Sugar/ bonbons |  |  |  |
| 12 | Beverages | 88 | Lipton tea | 28 | Lipton tea |  |
|  |  | 89 | Nescafé | 29 | Nescafé |  |
|  |  | 90 | Cola, fanta, sprite (sucré) | 30 | Beverages | Food items combined |
|  |  | 91 | Orange juice |  |  |  |
|  |  | 92 | Tamarind juice |  |  |  |
