# Supplementary figures and images for: Dietary habits associated with growth development of children aged < 5 years in the Nouna Health and Demographic Surveillance System, Burkina Faso
Source: Nutr J. 2020 Aug 9;19:81. doi: 10.1186/s12937-020-00591-3 (PMC7416397; doi:10.1186/s12937-020-00591-3)

Suppl. Figure 1: Scree plot of eigenvalues after factor analysis to derive dietary patterns


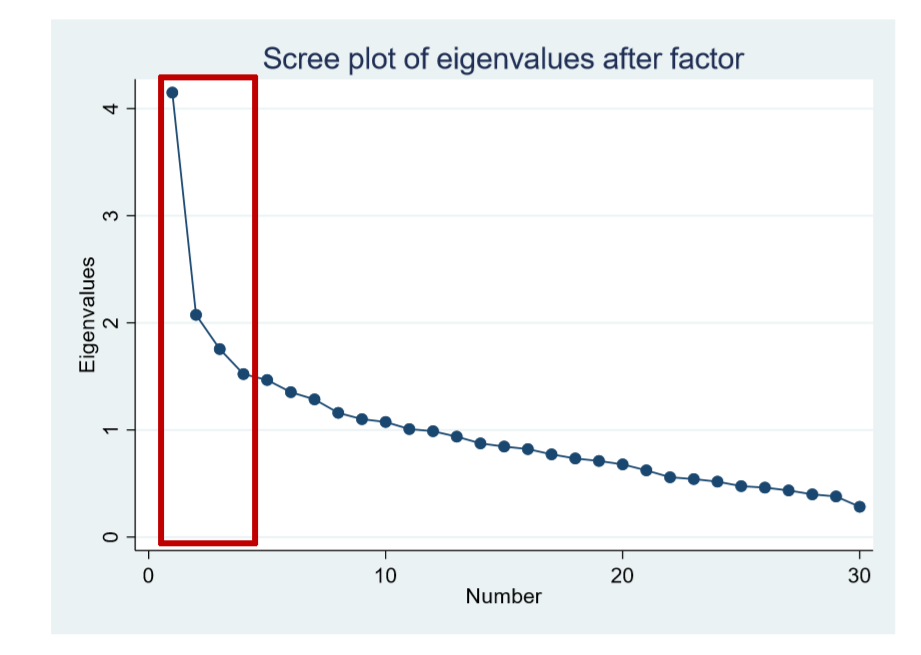

Supplement: Supplementary file 2 — Additional file 2:. Figure 1 Scree plot of eigenvalues after factor analysis to derive dietary patterns. [file 12937_2020_591_MOESM2_ESM.docx]
