# Supplementary material for: Dietary habits associated with growth development of children aged < 5 years in the Nouna Health and Demographic Surveillance System, Burkina Faso
Source: Nutr J. 2020 Aug 9;19:81. doi: 10.1186/s12937-020-00591-3 (PMC7416397; doi:10.1186/s12937-020-00591-3)
Supplement: Supplementary file 3 — Additional file 3: Table 2 Rotated factor loadings of food items for the four identified dietary pattern scores among children aged < 5 years (n = 514) in the Nouna HDSS. * Factor loading scores above 0.40 indicate relevant contribution to the pattern score. [file 12937_2020_591_MOESM3_ESM.docx]

Table 2: Rotated factor loadings of food items for the four identified dietary pattern scores among children aged < 5 years (n=514) in the Nouna HDSS

| **Food groups** | **Dietary pattern 1** | **Dietary pattern 2** | **Dietary pattern 3** | **Dietary pattern 4** |
| --- | --- | --- | --- | --- |
|  | **Leaves-based** | **Beans and poultry-based** | **Maize and fish-based** | **Millet and meat-based** |
| Cabbage | **0.63*** | 0.10 | -0.02 | -0.05 |
| Peanuts | **0.63*** | 0.27 | 0.03 | 0.02 |
| Vitamin A-rich leaves | **0.61*** | 0.09 | -0.28 | -0.02 |
| Onions | **0.56*** | 0.03 | 0.18 | 0.14 |
| African locust bean | **0.43*** | -0.04 | 0.35 | 0.18 |
| Couscous | **0.42*** | -0.09 | 0.04 | -0.22 |
| Cowpea beans | -0.04 | **0.63*** | 0.06 | 0.08 |
| Bread | 0.13 | **0.61*** | 0.17 | -0.03 |
| Poultry | 0.14 | **0.59*** | -0.17 | 0.00 |
| Pasta | 0.37 | **0.49*** | 0.10 | 0.07 |
| Sweets | 0.24 | **0.49*** | 0.20 | 0.09 |
| Milk powder | -0.04 | **0.46*** | 0.06 | 0.12 |
| Fish | 0.05 | 0.15 | **0.62*** | 0.15 |
| Okra | -0.15 | -0.10 | **0.58*** | 0.05 |
| Eggplant | 0.13 | 0.20 | **0.55*** | -0.15 |
| Maize | -0.13 | 0.13 | **0.46*** | **-0.52*** |
| Oils and fats | 0.34 | -0.23 | **0.43*** | 0.25 |
| Millet | -0.06 | 0.05 | 0.08 | **0.75*** |
| Flesh meat | 0.34 | 0.29 | 0.07 | **0.46*** |
| Rice | 0.36 | 0.26 | 0.02 | 0.35 |
| Animal milk | 0.36 | 0.04 | 0.21 | 0.31 |
| Lipton tea | 0.27 | 0.17 | 0.13 | 0.09 |
| Sorghum | 0.24 | -0.16 | -0.27 | -0.02 |
| Eggs | 0.24 | 0.32 | 0.00 | -0.05 |
| Fruits | 0.19 | 0.26 | -0.06 | -0.14 |
| Cassava | 0.16 | 0.18 | 0.30 | -0.25 |
| Beverages | 0.14 | 0.35 | 0.09 | -0.15 |
| Nescafé | -0.02 | 0.11 | 0.08 | 0.34 |
| Tomatoes | -0.10 | 0.20 | 0.39 | -0.01 |
| Mother's milk | -0.19 | -0.02 | -0.05 | 0.04 |
| **Explained variance** | **9.82%** | **8.79%** | **7.28%** | **5.79%** |

* Factor loading scores above 0.40 indicate relevant contribution to the pattern score.
