# Supplementary material for: Dietary habits associated with growth development of children aged < 5 years in the Nouna Health and Demographic Surveillance System, Burkina Faso
Source: Nutr J. 2020 Aug 9;19:81. doi: 10.1186/s12937-020-00591-3 (PMC7416397; doi:10.1186/s12937-020-00591-3)
Supplement: Supplementary file 4 — Additional file 4:. Table 3 Percentage of 523 children aged < 5 years who consume FFQ-derived food groups according to the eating time of the day. [file 12937_2020_591_MOESM4_ESM.docx]

Table 3: Percentage of 523 children aged < 5 years who consume FFQ-derived food groups according to the eating time of the day

|  | **FG** | **Obs.** | **Early morning** | **Late morning** | **Noon** | **Afternoon** | **Evening** | **Night** |
| --- | --- | --- | --- | --- | --- | --- | --- | --- |
|  |  |  | <9am | 9am-12pm | 12-2pm | 2-5pm | 5-8pm | >8pm |
| N=523 |  |  | 389 | 315 | 403 | 283 | 477 | 104 |
| % |  |  | 74.38 | 60.23 | 77.06 | 54.11 | 91.20 | 19.89 |
| **Food groups** |  |  |  |  |  |  |  |  |
| Cereales, starchy roots, tubers and their products | 1 | 1,634 | 21.36 | 15.42 | 21.54 | 13.04 | 26.19 | 2.45 |
| Pulses, nuts, seeds and their products | 2 | 193 | 15.54 | 13.47 | 29.53 | 11.40 | 26.42 | 3.63 |
| Vegetables | 3 | 536 | 15.67 | 17.35 | 23.32 | 12.50 | 28.92 | 2.24 |
| Fruits | 4 | 79 | 20.25 | 11.39 | 25.32 | 6.33 | 32.91 | 3.80 |
| Vitamin A rich fruits and vegetables | 5 | 764 | 15.58 | 15.71 | 24.35 | 10.73 | 30.63 | 3.01 |
| Flesh meat | 6 | 72 | 11.11 | 19.44 | 27.78 | 11.11 | 30.56 | 0.00 |
| Fish and seafood | 7 | 75 | 12.00 | 16.00 | 36.00 | 4.00 | 30.67 | 1.33 |
| Oils and fats | 8 | 35 | 8.57 | 22.86 | 22.86 | 11.43 | 34.29 | 0.00 |
| Milk and milk products | 9 | 484 | 19.83 | 19.21 | 14.46 | 17.98 | 14.67 | 13.84 |
| Eggs | 10 | 0 | 0.00 | 0.00 | 0.00 | 0.00 | 0.00 | 0.00 |
