# Supplementary material for: Dietary habits associated with growth development of children aged < 5 years in the Nouna Health and Demographic Surveillance System, Burkina Faso
Source: Nutr J. 2020 Aug 9;19:81. doi: 10.1186/s12937-020-00591-3 (PMC7416397; doi:10.1186/s12937-020-00591-3)
Supplement: Supplementary file 5 — Additional file 5:. Table 4 Percentage of 523 children aged < 5 years who consume FFQ-derived food groups according to age group. [file 12937_2020_591_MOESM5_ESM.docx]

Table 4: Percentage of 523 children aged < 5 years who consume FFQ-derived food groups according to age group

| **Food group** | | **All children** | | **% of children by age in months** | | | |
| --- | --- | --- | --- | --- | --- | --- | --- |
|  |  | **N** | **8-59** | **8-23** | **24-35** | **36-47** | **48-59** |
| 1 | Cereals, starchy roots, tubers and their products | 508 | 98.64 | 25.00 | 22.24 | 26.57 | 26.18 |
| 2 | Pulses, nuts, seeds and their products | 439 | 85.24 | 23.69 | 23.92 | 25.74 | 26.65 |
| 3 | Vegetables | 404 | 78.45 | 22.03 | 23.02 | 26.98 | **27.97** |
| 4 | Fruits | 316 | 61.36 | 21.52 | 22.15 | 28.16 | 28.16 |
| 5 | Vitamin A rich fruits and vegetables | 484 | 93.98 | 23.35 | 22.73 | 27.48 | 26.45 |
| 6 | Flesh meat | 302 | 58.64 | 18.54 | 21.85 | **31.46** | **28.15** |
| 7 | Fish and seafood | 251 | 48.74 | 21.91 | 23.11 | **26.29** | **28.69** |
| 8 | Oils and fats | 475 | 92.23 | 22.95 | 23.37 | 26.53 | 27.16 |
| 9 | Milk and milk products | 278 | 53.98 | **41.37** | 17.27 | 21.58 | 19.78 |
| 10 | Eggs | 53 | 10.29 | 20.75 | 18.87 | **30.19** | **30.19** |
| 11 | Sweets | 366 | 71.07 | 23.22 | 24.04 | 28.96 | 23.77 |
| 12 | Beverages | 229 | 44.47 | 20.09 | 24.89 | 27.95 | 27.07 |
