# Supplementary material for: Dietary habits associated with growth development of children aged < 5 years in the Nouna Health and Demographic Surveillance System, Burkina Faso
Source: Nutr J. 2020 Aug 9;19:81. doi: 10.1186/s12937-020-00591-3 (PMC7416397; doi:10.1186/s12937-020-00591-3)
Supplement: Supplementary file 6 — Additional file 6: Table 5 Characteristics and frequencies of food intake of children aged < 5 years (n = 514) across tertiles of dietary pattern scores: leaves-based diet, beans-based diet, maize-based diet, and millet-based diet. * p-value < 0.05, ** p-value < 0.01, *** p-value < 0.001. * p-value < 0.05, ** p-value < 0.01, *** p-value < 0.001. * p-value < 0.05, ** p-value < 0.01, *** p-value < 0.001. * p-value < 0.05, ** p-value < 0.01, *** p-value < 0.001. [file 12937_2020_591_MOESM6_ESM.docx]

Table 5: Characteristics and frequencies of food intake of children aged < 5 years (n=514) across tertiles of dietary pattern scores: leaves-based diet, beans-based diet, maize-based diet, and millet-based diet

| **Variables** | | **Tertile 1** | | **Tertile 2** | | **Tertile 3** | | **P-value** |
| --- | --- | --- | --- | --- | --- | --- | --- | --- |
|  |  | Mean / % | (SD / SE) | Mean / % | (SD / SE) | Mean / % | (SD / SE) | Trend |
| **Leaves-based diet** |  |  |  |  |  |  |  |  |
| N |  | 172 | | 171 | | 171 | |  |
| Tertile scores |  | 5.69 | (2.85) | 11.69 | (1.63) | 19.72 | (4.64) |  |
|  |  |  |  |  |  |  |  |  |
| **Demographics** |  |  |  |  |  |  |  |  |
| Child's sex | Boys | 30.59 | (0.03) | 36.08 | (0.03) | 33.33 | (0.03) | 0.419 |
|  | Girls | 36.29 | (0.03) | 30.50 | (0.03) | 33.20 | (0.03) |  |
| Child's age | Months | 32.97 | (15.34) | 36.28 | (13.04) | 38.37 | (12.47) | 0.001** |
|  |  |  |  |  |  |  |  |  |
| **Frequency of food intake** |  | Median | IQR | Median | IQR | Median | IQR |  |
| African locust beans |  | 0 | 3 | 4 | 6 | 7 | 3 | 0.000*** |
| Animal milk |  | 0 | 0 | 0 | 2 | 1 | 3 | 0.000*** |
| Vitamin A-rich leaves |  | 4 | 4 | 7 | 4 | 10 | 6 | 0.000*** |
| Beverages |  | 0 | 0 | 0 | 0 | 0 | 0 | 0.000*** |
| Bread |  | 0 | 0 | 0 | 0 | 0 | 0 | 0.000*** |
| Cabbage |  | 0 | 0 | 0 | 0 | 0 | 1 | 0.000*** |
| Cassava |  | 0 | 0 | 0 | 0 | 0 | 0 | 0.003** |
| Poultry |  | 0 | 0 | 0 | 0 | 0 | 0 | 0.000*** |
| Couscous |  | 0 | 0 | 0 | 0 | 0 | 0 | 0.000*** |
| Cowpea beans |  | 0 | 0 | 0 | 0 | 0 | 1 | 0.000*** |
| Eggplant |  | 0 | 0 | 0 | 0 | 0 | 1 | 0.000*** |
| Eggs |  | 0 | 0 | 0 | 0 | 0 | 0 | 0.000*** |
| Fish |  | 0 | 2 | 1 | 3 | 1 | 3 | 0.003** |
| Lipton tea |  | 0 | 0 | 0 | 3 | 0 | 7 | 0.000*** |
| Maize |  | 5 | 7 | 2 | 7 | 3 | 6 | 0.001** |
| Red meat |  | 0 | 0 | 1 | 2 | 1 | 1 | 0.000*** |
| Milk powder |  | 0 | 0 | 0 | 0 | 0 | 0 | 0.001** |
| Millet |  | 0 | 2.5 | 1 | 4 | 1 | 4 | 0.001** |
| Maternal milk |  | 0 | 7 | 0 | 0 | 0 | 0 | 0.000*** |
| Nescafé |  | 0 | 0 | 0 | 0 | 0 | 0 | 0.013* |
| Oils and fats |  | 5 | 6 | 7 | 2 | 7 | 2 | 0.000*** |
| Okra |  | 2 | 3 | 2 | 3 | 1 | 3 | 0.004** |
| Onions |  | 0 | 0 | 0 | 1 | 1 | 3 | 0.000*** |
| Pasta |  | 0 | 0 | 0 | 0 | 0 | 2 | 0.000*** |
| Peanuts |  | 0 | 0.5 | 0 | 2 | 3 | 3 | 0.000*** |
| Rice |  | 0.5 | 1 | 1 | 2 | 2 | 2 | 0.000*** |
| Fruits |  | 0 | 6.5 | 4 | 7 | 7 | 5 | 0.000*** |
| Sorghum |  | 0 | 3 | 1 | 5 | 2 | 6 | 0.000*** |
| Sweets |  | 1 | 2 | 2 | 3 | 3 | 4 | 0.000*** |
| Tomatoes |  | 0 | 0 | 0 | 0 | 0 | 0 | 0.347 |

* p-value <0.05, ** p-value <0.01, *** p-value <0.001

| **Variables** | | **Tertile 1** | | **Tertile 2** | | **Tertile 3** | | **P-value** |
| --- | --- | --- | --- | --- | --- | --- | --- | --- |
|  |  | Mean / % | (SD / SE) | Mean / % | (SD / SE) | Mean / % | (SD / SE) | Trend |
| **Beans and poultry-based diet** |  |  |  |  |  |  |  |  |
| N |  | 172 | | 171 | | 171 | |  |
| Tertile scores |  | 0.96 | (1.03) | 3.75 | (0.83) | 8.44 | (3.42) |  |
|  |  |  |  |  |  |  |  |  |
| **Demographics** |  |  |  |  |  |  |  |  |
| Child's sex | Boys | 32.55 | (0.03) | 31.37 | (0.03) | 36.08 | (0.03) | 0.306 |
|  | Girls | 34.36 | (0.03) | 35.14 | (0.03) | 30.50 | (0.03) |  |
| Child's age | Months | 32.99 | (14.80) | 37.04 | (13.85) | 37.59 | (12.58) | 0.003** |
|  |  |  |  |  |  |  |  |  |
| **Frequency of food intake** |  | Median | IQR | Median | IQR | Median | IQR |  |
| African locust beans |  | 2 | 5 | 4 | 7 | 5 | 5 | 0.000*** |
| Animal milk |  | 0 | 0 | 0 | 2 | 1 | 3 | 0.000*** |
| Vitamin A-rich leaves |  | 6 | 4.5 | 6 | 5 | 8 | 5 | 0.000*** |
| Beverages |  | 0 | 0 | 0 | 0 | 0 | 0 | 0.000*** |
| Bread |  | 0 | 0 | 0 | 0 | 0 | 1 | 0.000*** |
| Cabbage |  | 0 | 0 | 0 | 0 | 0 | 1 | 0.000*** |
| Cassava |  | 0 | 0 | 0 | 0 | 0 | 0 | 0.000*** |
| Poultry |  | 0 | 0 | 0 | 0 | 0 | 1 | 0.000*** |
| Couscous |  | 0 | 0 | 0 | 0 | 0 | 0 | 0.000*** |
| Cowpea beans |  | 0 | 0 | 0 | 0 | 0 | 1 | 0.000*** |
| Eggplant |  | 0 | 0 | 0 | 0 | 0 | 2 | 0.000*** |
| Eggs |  | 0 | 0 | 0 | 0 | 0 | 0 | 0.000*** |
| Fish |  | 0 | 1 | 1 | 3 | 2 | 3 | 0.000*** |
| Lipton tea |  | 0 | 0 | 0 | 2 | 0 | 7 | 0.000*** |
| Maize |  | 2 | 6 | 4 | 7 | 5 | 6 | 0.000*** |
| Red meat |  | 0 | 1 | 0 | 2 | 1 | 1 | 0.000*** |
| Milk powder |  | 0 | 0 | 0 | 0 | 0 | 0 | 0.000*** |
| Millet |  | 0 | 3 | 0 | 4 | 0 | 5 | 0.010* |
| Maternal milk |  | 0 | 7 | 0 | 0 | 0 | 0 | 0.007** |
| Nescafé |  | 0 | 0 | 0 | 0 | 0 | 0 | 0.000*** |
| Oils and fats |  | 7 | 4 | 7 | 3 | 7 | 3 | 0.392 |
| Okra |  | 1 | 3 | 2 | 3 | 2 | 3 | 0.993 |
| Onions |  | 0 | 0 | 0 | 1 | 0 | 3 | 0.000*** |
| Pasta |  | 0 | 0 | 0 | 0 | 0 | 2 | 0.000*** |
| Peanuts |  | 0 | 1 | 1 | 2 | 3 | 3 | 0.000*** |
| Rice |  | 1 | 1 | 1 | 2 | 2 | 2 | 0.000*** |
| Fruits |  | 0 | 4 | 5 | 7 | 7 | 9 | 0.000*** |
| Sorghum |  | 2 | 5 | 1 | 5 | 0 | 3 | 0.029* |
| Sweets |  | 0 | 2 | 2 | 3 | 4 | 4 | 0.000*** |
| Tomatoes |  | 0 | 0 | 0 | 0 | 0 | 1 | 0.000*** |

* p-value <0.05, ** p-value <0.01, *** p-value <0.001

| **Variables** | | **Tertile 1** | | **Tertile 2** | | **Tertile 3** | | **P-value** |
| --- | --- | --- | --- | --- | --- | --- | --- | --- |
|  |  | Mean / % | (SD / SE) | Mean / % | (SD / SE) | Mean / % | (SD / SE) | Trend |
| **Maize and fish-based diet** |  |  |  |  |  |  |  |  |
| N |  | 172 | | 171 | | 171 | |  |
| Tertile scores |  | 1.58 | (2.28) | 6.46 | (1.20) | 12.23 | (3.04) |  |
|  |  |  |  |  |  |  |  |  |
| **Demographics** |  |  |  |  |  |  |  |  |
| Child's sex | Boys | 30.98 | (0.03) | 38.82 | (0.03) | 30.20 | (0.03) | 0.871 |
|  | Girls | 35.91 | (0.03) | 27.80 | (0.03) | 36.29 | (0.03) |  |
| Child's age | Months | 32.98 | (14.85) | 37.85 | (12.87) | 36.79 | (13.52) | 0.015* |
|  |  |  |  |  |  |  |  |  |
| **Frequency of food intake** |  | Median | IQR | Median | IQR | Median | IQR |  |
| African locust beans |  | 0 | 6 | 3 | 7 | 7 | 4 | 0.000*** |
| Animal milk |  | 0 | 1 | 0 | 2 | 0 | 3 | 0.000*** |
| Vitamin A-rich leaves |  | 8 | 4.5 | 7 | 4 | 5 | 4 | 0.000*** |
| Beverages |  | 0 | 0 | 0 | 0 | 0 | 0 | 0.118 |
| Bread |  | 0 | 0 | 0 | 0 | 0 | 0 | 0.000*** |
| Cabbage |  | 0 | 0 | 0 | 0 | 0 | 0 | 0.467 |
| Cassava |  | 0 | 0 | 0 | 0 | 0 | 0 | 0.000*** |
| Poultry |  | 0 | 0 | 0 | 0 | 0 | 0 | 0.884 |
| Couscous |  | 0 | 0 | 0 | 0 | 0 | 0 | 0.520 |
| Cowpea beans |  | 0 | 0 | 0 | 1 | 0 | 1 | 0.005** |
| Eggplant |  | 0 | 0 | 0 | 0 | 0 | 2 | 0.000*** |
| Eggs |  | 0 | 0 | 0 | 0 | 0 | 0 | 0.089 |
| Fish |  | 0 | 0 | 0 | 2 | 3 | 4 | 0.000*** |
| Lipton tea |  | 0 | 0 | 0 | 3 | 0 | 3 | 0.000*** |
| Maize |  | 0 | 2 | 4 | 5 | 7 | 6 | 0.000*** |
| Red meat |  | 0 | 1 | 1 | 2 | 1 | 2 | 0.020* |
| Milk powder |  | 0 | 0 | 0 | 0 | 0 | 0 | 0.049* |
| Millet |  | 0 | 3 | 0 | 5 | 0 | 4 | 0.155 |
| Maternal milk |  | 0 | 7 | 0 | 0 | 0 | 0 | 0.130 |
| Nescafé |  | 0 | 0 | 0 | 0 | 0 | 0 | 0.070 |
| Oils and fats |  | 4 | 6 | 7 | 3 | 7 | 0 | 0.000*** |
| Okra |  | 0 | 1 | 2 | 3 | 3 | 2 | 0.000*** |
| Onions |  | 0 | 0 | 0 | 2 | 0 | 3 | 0.000*** |
| Pasta |  | 0 | 0 | 0 | 1 | 0 | 1 | 0.083 |
| Peanuts |  | 0 | 2.5 | 1 | 3 | 1 | 3 | 0.087 |
| Rice |  | 1 | 2 | 1 | 2 | 1 | 2 | 0.025* |
| Fruits |  | 4.5 | 7 | 4 | 7 | 3 | 7 | 0.364 |
| Sorghum |  | 4 | 7 | 0 | 3 | 0 | 3 | 0.000*** |
| Sweets |  | 1 | 2 | 2 | 3 | 2 | 5 | 0.000*** |
| Tomatoes |  | 0 | 0 | 0 | 0 | 0 | 2 | 0.000*** |

* p-value <0.05, ** p-value <0.01, *** p-value <0.001

| **Variables** | | **Tertile 1** | | **Tertile 2** | | **Tertile 3** | | **P-value** |
| --- | --- | --- | --- | --- | --- | --- | --- | --- |
|  |  | Mean / % | (SD / SE) | Mean / % | (SD / SE) | Mean / % | (SD / SE) | Trend |
| **Millet and meat-based diet** |  |  |  |  |  |  |  |  |
| N |  | 172 | | 171 | | 171 | |  |
| Tertile scores |  | -1.82 | (2.02) | 2.60 | (1.05) | 7.88 | (2.74) |  |
|  |  |  |  |  |  |  |  |  |
| **Demographics** |  |  |  |  |  |  |  |  |
| Child's sex | Boys | 31.37 | (0.03) | 35.29 | (0.03) | 33.33 | (0.03) | 0.553 |
|  | Girls | 35.52 | (0.03) | 31.27 | (0.03) | 33.20 | (0.03) |  |
| Child's age | Months | 35.78 | (14.03) | 34.95 | (13.83) | 36.87 | (13.86) | 0.425 |
|  |  |  |  |  |  |  |  |  |
| **Frequency of food intake** |  | Median | IQR | Median | IQR | Median | IQR |  |
| African locust beans |  | 1 | 7 | 3 | 7 | 6 | 4 | 0.000*** |
| Animal milk |  | 0 | 0 | 0 | 2 | 1 | 3 | 0.000*** |
| Vitamin A-rich leaves |  | 6 | 5 | 7 | 5 | 7 | 4 | 0.024* |
| Beverages |  | 0 | 0 | 0 | 0 | 0 | 0 | 0.514 |
| Bread |  | 0 | 0 | 0 | 0 | 0 | 0 | 0.966 |
| Cabbage |  | 0 | 0 | 0 | 0 | 0 | 0 | 0.007** |
| Cassava |  | 0 | 0 | 0 | 0 | 0 | 0 | 0.025* |
| Poultry |  | 0 | 0 | 0 | 0 | 0 | 0 | 0.869 |
| Couscous |  | 0 | 0 | 0 | 0 | 0 | 0 | 0.878 |
| Cowpea beans |  | 0 | 0.5 | 0 | 1 | 0 | 0 | 0.946 |
| Eggplant |  | 0 | 0.5 | 0 | 0 | 0 | 0 | 0.346 |
| Eggs |  | 0 | 0 | 0 | 0 | 0 | 0 | 0.388 |
| Fish |  | 0 | 2 | 0 | 2 | 1 | 3 | 0.002** |
| Lipton tea |  | 0 | 0 | 0 | 3 | 0 | 3 | 0.003** |
| Maize |  | 7 | 5 | 3 | 6 | 1 | 3 | 0.000*** |
| Red meat |  | 0 | 1 | 1 | 2 | 1 | 2 | 0.000*** |
| Milk powder |  | 0 | 0 | 0 | 0 | 0 | 0 | 0.019* |
| Millet |  | 0 | 0 | 0 | 2 | 5 | 4 | 0.000*** |
| Maternal milk |  | 0 | 0 | 0 | 0 | 0 | 0 | 0.969 |
| Nescafé |  | 0 | 0 | 0 | 0 | 0 | 0 | 0.000*** |
| Oils and fats |  | 5 | 5 | 7 | 3 | 7 | 1 | 0.000*** |
| Okra |  | 1 | 3 | 1 | 3 | 2 | 3 | 0.026* |
| Onions |  | 0 | 0 | 0 | 1 | 0 | 3 | 0.000*** |
| Pasta |  | 0 | 0 | 0 | 1 | 0 | 1 | 0.002** |
| Peanuts |  | 0 | 2 | 1 | 3 | 2 | 4 | 0.000*** |
| Rice |  | 1 | 1 | 1 | 1 | 2 | 1 | 0.000*** |
| Fruits |  | 4 | 7 | 5 | 7 | 3 | 7 | 0.152 |
| Sorghum |  | 0 | 3 | 3 | 6 | 1 | 4 | 0.032* |
| Sweets |  | 1 | 3 | 2 | 2 | 2 | 3 | 0.000*** |
| Tomatoes |  | 0 | 0 | 0 | 0 | 0 | 0 | 0.924 |

* p-value <0.05, ** p-value <0.01, *** p-value <0.001
