# Supplementary material for: Dietary habits associated with growth development of children aged < 5 years in the Nouna Health and Demographic Surveillance System, Burkina Faso
Source: Nutr J. 2020 Aug 9;19:81. doi: 10.1186/s12937-020-00591-3 (PMC7416397; doi:10.1186/s12937-020-00591-3)
Supplement: Supplementary file 7 — Additional file 7:. Table 6 Sensitivity analyses of associations of DDS, FVS, and four DPS with stunting (HAZ < − 2) of children aged < 5 years (n = 514) in the Nouna HDSS. * p-value < 0.05, ** p-value < 0.01, *** p-value < 0.001. [file 12937_2020_591_MOESM7_ESM.docx]

Table 6: Sensitivity analyses of associations of DDS, FVS, and four DPS with stunting (HAZ < -2) of children aged < 5 years (n=514) in the Nouna HDSS

| **Stunting (HAZ < -2)** | | **Per 1 score-point increase** | | |  | **Tercile 1** | **Tercile 2** | | **Tercile 3** | |
| --- | --- | --- | --- | --- | --- | --- | --- | --- | --- | --- |
|  |  | PR | 95% CI | p-value trend |  |  | PR | 95% CI | PR | 95% CI |
| **Dietary Diversity Score (DDS)** | |  |  |  |  |  |  |  |  |  |
| Model 1 | Crude model: stunting | 1.03 | 1.01, 1.06 | 0.003** |  | Ref. | 1.45 | 1.32, 1.60 | 1.07 | 0.98, 1.17 |
| Model 2 | Model 1 + demographics | 1.01 | 0.99, 1.03 | 0.365 |  | Ref. | 1.35 | 1.23, 1.49 | 1.00 | 0.91, 1.09 |
| Model 3 | Model 2 + socio-economics + clinical | 1.03 | 1.01, 1.06 | 0.014* |  | Ref. | 1.20 | 1.08, 1.32 | 1.11 | 1.00, 1.22 |
|  |  |  |  |  |  |  |  |  |  |  |
| **Food Variety Score (FVS)** | |  |  |  |  |  |  |  |  |  |
| Model 1 | Crude model: stunting | 1.02 | 1.02, 1.03 | 0.000*** |  | Ref. | 1.23 | 1.12, 1.35 | 1.31 | 1.19, 1.44 |
| Model 2 | Model 1 + demographics | 1.02 | 1.01, 1.03 | 0.000*** |  | Ref. | 1.18 | 1.07, 1.30 | 1.20 | 1.08, 1.33 |
| Model 3 | Model 2 + socio-economics + clinical | 1.03 | 1.02, 1.03 | 0.000*** |  | Ref. | 1.11 | 1.00, 1.23 | 1.27 | 1.13, 1.42 |
|  |  |  |  |  |  |  |  |  |  |  |
| **Leaves-based dietary pattern score** | |  |  |  |  |  |  |  |  |  |
| Model 1 | Crude model: stunting | 1.02 | 1.02, 1.03 | 0.000*** |  | Ref. | 1.01 | 0.92, 1.12 | 1.47 | 1.35, 1.61 |
| Model 2 | Model 1 + demographics | 1.02 | 1.01, 1.02 | 0.00*** |  | Ref. | 0.97 | 0.87, 1.07 | 1.35 | 1.21, 1.49 |
| Model 3 | Model 2 + socio-economics + clinical | 1.00 | 1.00, 1.01 | 0.426 |  | Ref. | 0.78 | 0.70, 0.87 | 1.05 | 0.93, 1.18 |
|  |  |  |  |  |  |  |  |  |  |  |
| **Beans and poultry-based dietary pattern score** | |  |  |  |  |  |  |  |  |  |
| Model 1 | Crude model: stunting | 1.00 | 0.99, 1.01 | 0.626 |  | Ref. | 0.79 | 0.72, 0.87 | 0.85 | 0.78, 0.93 |
| Model 2 | Model 1 + demographics | 1.00 | 0.99, 1.01 | 0.675 |  | Ref. | 0.79 | 0.72, 0.87 | 0.78 | 0.71, 0.86 |
| Model 3 | Model 2 + socio-economics + clinical | 1.02 | 1.01, 1.04 | 0.000*** |  | Ref. | 0.79 | 0.72, 0.87 | 0.89 | 0.80, 0.98 |
|  |  |  |  |  |  |  |  |  |  |  |
| **Maize and fish-based dietary pattern score** | |  |  |  |  |  |  |  |  |  |
| Model 1 | Crude model: stunting | 0.98 | 0.97, 0.99 | 0.000*** |  | Ref. | 0.96 | 0.87, 1.05 | 0.89 | 0.81, 0.97 |
| Model 2 | Model 1 + demographics | 0.99 | 0.98, 1.00 | 0.012* |  | Ref. | 1.03 | 0.94, 1.14 | 1.06 | 0.95, 1.17 |
| Model 3 | Model 2 + socio-economics + clinical | 0.99 | 0.98, 1.00 | 0.007** |  | Ref. | 1.24 | 1.12, 1.37 | 1.03 | 0.93, 1.15 |
|  |  |  |  |  |  |  |  |  |  |  |
| **Millet and meat-based dietary pattern score** | |  |  |  |  |  |  |  |  |  |
| Model 1 | Crude model: stunting | 1.01 | 1.00, 1.02 | 0.005** |  | Ref. | 1.14 | 1.03, 1.25 | 1.42 | 1.30, 1.56 |
| Model 2 | Model 1 + demographics | 1.01 | 1.00, 1.01 | 0.196 |  | Ref. | 1.01 | 0.92, 1.11 | 1.36 | 1.23, 1.50 |
| Model 3 | Model 2 + socio-economics + clinical | 0.98 | 0.97, 0.99 | 0.000*** |  | Ref. | 0.94 | 0.84, 1.04 | 1.08 | 0.96, 1.21 |

* p-value <0.05, ** p-value <0.01, *** p-value <0.001
