# Supplementary material for: Dietary habits associated with growth development of children aged < 5 years in the Nouna Health and Demographic Surveillance System, Burkina Faso
Source: Nutr J. 2020 Aug 9;19:81. doi: 10.1186/s12937-020-00591-3 (PMC7416397; doi:10.1186/s12937-020-00591-3)
Supplement: Supplementary file 8 — Additional file 8:. Table 7: Sensitivity analyses of associations of DDS, FVS and four DPS with wasting (WHZ < − 2) of children aged < 5 years (n = 514) in the Nouna HDSS. * p-value < 0.05, ** p-value < 0.01, *** p-value < 0.001. [file 12937_2020_591_MOESM8_ESM.docx]

Table 7: Sensitivity analyses of associations of DDS, FVS and four DPS with wasting (WHZ < -2) of children aged < 5 years (n=514) in the Nouna HDSS

| **Wasting (WHZ < -2)** | | **Per 1 score-point increase** | | |  | **Tercile 1** | **Tercile 2** | | **Tercile 3** | |
| --- | --- | --- | --- | --- | --- | --- | --- | --- | --- | --- |
|  |  | PR | 95% CI | p-value trend |  |  | PR | 95% CI | β-coef. | 95% CI |
| **Dietary Diversity Score (DDS)** | |  |  |  |  |  |  |  |  |  |
| Model 1 | Crude model: wasting | 0.90 | 0.87, 0.94 | 0.000*** |  | Ref. | 0.56 | 0.46, 0.69 | 0.66 | 0.56, 0.78 |
| Model 2 | Model 1 + demographics | 0.95 | 0.91, 0.99 | 0.023* |  | Ref. | 0.60 | 0.49, 0.74 | 0.73 | 0.61, 0.87 |
| Model 3 | Model 2 + socio-economics + clinical | 0.93 | 0.89, 0.97 | 0.001** |  | Ref. | 0.52 | 0.42, 0.65 | 0.60 | 0.49, 0.73 |
|  |  |  |  |  |  |  |  |  |  |  |
| **Food Variety Score (FVS)** | |  |  |  |  |  |  |  |  |  |
| Model 1 | Crude model: wasting | 0.94 | 0.93, 0.96 | 0.000*** |  | Ref. | 0.58 | 0.49, 0.69 | 0.50 | 0.42, 0.61 |
| Model 2 | Model 1 + demographics | 0.96 | 0.94, 0.98 | 0.000*** |  | Ref. | 0.59 | 0.49, 0.71 | 0.60 | 0.49, 0.73 |
| Model 3 | Model 2 + socio-economics + clinical | 0.95 | 0.94, 0.97 | 0.000*** |  | Ref. | 0.54 | 0.44, 0.65 | 0.56 | 0.45, 0.69 |
|  |  |  |  |  |  |  |  |  |  |  |
| **Leaves-based dietary pattern score** | |  |  |  |  |  |  |  |  |  |
| Model 1 | Crude model: wasting | 0.98 | 0.96, 0.99 | 0.000*** |  | Ref. | 1.52 | 1.27, 1.81 | 0.91 | 0.75, 1.11 |
| Model 2 | Model 1 + demographics | 0.99 | 0.98, 1.00 | 0.097 |  | Ref. | 1.77 | 1.47, 2.12 | 1.05 | 0.84, 1.31 |
| Model 3 | Model 2 + socio-economics + clinical | 0.98 | 0.96, 0.99 | 0.001*** |  | Ref. | 1.68 | 1.37, 2.06 | 0.83 | 0.65, 1.07 |
|  |  |  |  |  |  |  |  |  |  |  |
| **Beans and poultry-based dietary pattern score** | |  |  |  |  |  |  |  |  |  |
| Model 1 | Crude model: wasting | 0.92 | 0.90, 0.94 | 0.000*** |  | Ref. | 0.73 | 0.61, 0.87 | 0.72 | 0.60, 0.86 |
| Model 2 | Model 1 + demographics | 0.95 | 0.93, 0.97 | 0.000*** |  | Ref. | 0.84 | 0.70, 1.01 | 0.96 | 0.80, 1.17 |
| Model 3 | Model 2 + socio-economics + clinical | 0.94 | 0.92, 0.97 | 0.000*** |  | Ref. | 0.81 | 0.67, 0.98 | 0.91 | 0.74, 1.12 |
|  |  |  |  |  |  |  |  |  |  |  |
| **Maize and fish-based dietary pattern score** | |  |  |  |  |  |  |  |  |  |
| Model 1 | Crude model: wasting | 0.90 | 0.89, 0.92 | 0.000*** |  | Ref. | 0.50 | 0.42, 0.59 | 0.39 | 0.32, 0.47 |
| Model 2 | Model 1 + demographics | 0.92 | 0.90, 0.93 | 0.000*** |  | Ref. | 0.59 | 0.49, 0.71 | 0.44 | 0.36, 0.54 |
| Model 3 | Model 2 + socio-economics + clinical | 0.89 | 0.87, 0.91 | 0.000*** |  | Ref. | 0.51 | 0.41, 0.62 | 0.35 | 0.28, 0.44 |
|  |  |  |  |  |  |  |  |  |  |  |
| **Millet and meat-based dietary pattern score** | |  |  |  |  |  |  |  |  |  |
| Model 1 | Crude model: wasting | 1.00 | 0.99, 1.02 | 0.807 |  | Ref. | 0.93 | 0.78, 1.11 | 0.93 | 0.78, 1.11 |
| Model 2 | Model 1 + demographics | 1.00 | 0.99, 1.02 | 0.681 |  | Ref. | 0.85 | 0.71, 1.02 | 0.94 | 0.77, 1.14 |
| Model 3 | Model 2 + socio-economics + clinical | 0.96 | 0.94, 0.98 | 0.001** |  | Ref. | 0.72 | 0.59, 0.88 | 0.59 | 0.47, 0.75 |

* p-value <0.05, ** p-value <0.01, *** p-value <0.001
